# Interventions to Reduce Fear of Cancer Recurrence Among People With Cancer: Scoping Review

**DOI:** 10.2196/81579

**Published:** 2026-04-22

**Authors:** Niu Li, Cynthia Wensley, Lisa Reynolds, Wal Baraza, Tatiana Osorio Leyton, Andrew Jull

**Affiliations:** 1 School of Nursing, Faculty of Medicine and Health Science, University of Auckland, 85 Park Road, Grafton, Auckland, 1010, New Zealand, 64 0274679941; 2 Department of Psychological Medicine, University of Auckland, Auckland, New Zealand; 3 Department of Surgery, Faculty of science, University of Auckland, Auckland, New Zealand; 4 Department of Surgery, Auckland City Hospital, Auckland, New Zealand; 5 Centre for Translational Research in Health, University of Auckland, Auckland, New Zealand

**Keywords:** fear of cancer recurrence, fear of progression, cancer survivor, psychological interventions, cancer survivors, scoping review, mobile health interventions, mental health, digital health intervention

## Abstract

**Background:**

Fear of cancer recurrence (FCR) is prevalent among cancer survivors, affecting between 39% and 97% of patients. FCR is associated with impaired concentration, sleep disturbances, decreased quality of life, and increased psychological distress and health care use. To date, the literature lacks a review that summarizes the breadth of psychological interventions available for reducing fear of recurrence.

**Objective:**

This review aims to identify and summarize the evidence on psychological interventions for addressing FCR across all cancers.

**Methods:**

The Joanna Briggs Institute method for scoping reviews guided the processes, and we reported the review following the PRISMA-ScR (Preferred Reporting Items for Systematic Reviews and Meta-Analyses extension for Scoping Reviews) checklist. We searched 5 databases (CINAHL, PsycInfo, the Cochrane Central Register of Controlled Trials, Embase, and MEDLINE) and 2 gray literature sources (ProQuest Dissertations & Theses Global and the World Health Organization International Clinical Trials Registry). Eligible studies included adults (≥18 years) diagnosed with cancer and evaluated psychological interventions aimed at reducing FCR. Data extraction captured study characteristics, intervention details, outcome effectiveness, and follow-up durations. We synthesized the findings using descriptive summaries and narrative analysis.

**Results:**

Overall, 5131 articles were screened, and 122 were included in this review; 48 (39.3%) involved patients with breast cancer, 47 (38.5%) focused on patients with multiple cancer types; over half of the studies (n=64, 52.5%) were randomized controlled trials. Only 28 (23%) studies explicitly reported the definition of FCR. Eighteen different measurement tools were used. Blended interventions (different combinations of cognitive behavioral therapy, mindfulness, acceptance and commitment therapy, and other strategies) formed the largest intervention category (n=38, 31.1%), followed by cognitive behavioral therapy interventions (n=26, 21.3%) and mindfulness-based interventions (n=24, 19.7%). Of the included studies, 104 (85.2%) demonstrated significant reductions in FCR. Most interventions were delivered face-to-face by disciplinary specialists (n=75, 61.5%), while some were delivered remotely (n=34, 27.9%), with the majority of these delivered via the website (n=18, 52.9%). Follow-up duration ranged from postintervention to 3 years.

**Conclusions:**

FCR has been the focus of an increasing number of studies since 2009, with the majority being randomized controlled trials. Most interventions are delivered face-to-face and rely on trained specialists. Most have had statistically significant results. However, the included studies demonstrated heterogeneity in terms of delivery, duration, and dose, requiring cautious interpretation of intervention effects. Future research should develop consistent guidelines to standardize the definition of FCR, the measurement tools used, and the timing of follow-up assessments. Long-term follow-up data are needed to evaluate the sustained effects.

## Introduction

In 1981, Northouse first introduced the concept of fear of cancer recurrence (FCR) to describe the concerns of cancer patients in remission who feared that their cancer might return [1]. FCR is defined as fear, worry, or concern about the possibility of cancer returning [2]. FCR arises when cancer-related cues or bodily sensations are interpreted as signs of threat. These threat appraisals, combined with difficulties managing distress, can lead to patterns of hypervigilance, worry, reassurance seeking, or avoidance [3-5]. In addition, the idea that staying alert prevents recurrence further maintains persistent monitoring and rumination. Ongoing uncertainty about symptoms or the course of illness can amplify anxiety and contribute to the persistence of FCR over time [5,6].

Previous studies report that between 39% and 97% of cancer survivors experience FCR, with 22% to 87% experiencing moderate to high levels of fear [7]. This wide range may be due to heterogeneity in cancer types and survivorship stages, differences in measurement instruments, and variation in the timing of assessment [7]. It is common for cancer survivors to experience short-term or low levels of FCR. In some cases, such fear may be protective by increasing vigilance to bodily symptoms and promoting positive health behaviors, such as attending regular follow-up appointments and maintaining a healthy lifestyle [8]. However, when it becomes persistent and severe, it can interfere with a person’s mental health and daily functioning [9,10]. FCR may disrupt concentration, decision-making, sleep, and social functioning [11,12]. It also decreases overall well-being and quality of life and increases psychological distress and resistance to follow-up appointments [13-15]. High levels of FCR are significantly associated with a range of psychological comorbidities, including depression, anxiety, impaired emotional functioning, and insomnia [6]. FCR is linked to frequent checkups and additional examinations [16], and high levels can increase health care use, particularly in primary care [17,18]. FCR may also significantly delay return to work or even lead to premature withdrawal from employment [19]. If untreated, FCR can continue unabated throughout a patient’s survival journey [20,21]. In a large cohort study, 44%‐56% of patients continued to report clinical levels of FCR up to 18 months post surgery [22].

To date, only one scoping review has summarized psychological interventions for FCR [23]. However, it was solely focused on cognitive behavioral–based approaches [23]. In our review, we aim to map studies of the psychological interventions designed to reduce FCR in people with cancer, detailing the study characteristics, intervention details, outcome measurement, and follow-up durations to provide an overview of the interventions and highlight evidence gaps. These insights could inform the development of potential strategies to address FCR in specific populations.

## Methods

### Study Design

The review followed the Joanna Briggs Institute method for scoping reviews [24] and was reported by the PRISMA-ScR (Preferred Reporting Items for Systematic Reviews and Meta-Analyses extension for Scoping Reviews) checklist [25]. We preregistered the scoping review protocol on the Open Science Framework (registration number: 29YA3) [26].

### Review Objective

The primary objective of this scoping review is to map and summarize the evidence on the effects of psychological interventions designed to reduce FCR in people with cancer.

The scoping review addressed the following questions:

1. How has FCR been defined?
2. What instruments have been used to measure FCR?
3. What psychological interventions have been investigated to reduce FCR?
4. What are the intervention characteristics (types, setting, duration of intervention content, mode of delivery, personnel involved, and number of sessions)?
5. What is the effect of interventions in reducing FCR?
6. What is the follow-up duration of interventions addressing FCR?

### Eligibility Criteria

We used the population/participant-concept-context framework [24] to define eligibility.

- Population: studies focused on FCR in all people who have been histologically and clinically diagnosed with cancer, aged 18 years or older at diagnosis.
- Concept: any type of intervention with a psychological component aiming to reduce FCR. There were no limitations on the intervention’s duration, frequency, delivery method, or type of provider.
- Context: interventions provided in any type of setting.

### Type of Evidence Sources

We included any quantitative study design that reported data relevant to the research questions, including randomized controlled trials (RCTs), quasi-experimental studies, nonrandomized intervention studies, observational studies, and mixed methods studies with extractable intervention data. Review articles were excluded to avoid data duplication, but the reference lists were screened for relevant studies. We also excluded ongoing trials and protocol papers.

### Search Strategy

The search strategy was initially focused on colorectal cancer, but due to an insufficient number of studies, it was broadened to include all people with cancer. The search strategy was developed based on three key concepts: (1) neoplasms, including synonyms such as neoplas*, cancer, carcinom*, malignant*, tumor*, and oncolog*; (2) fear, with related terms including anxiety, worr*, concern, and distress; and (3) recurrence, with associated terms such as relapse, progress*, reappearance, and return. These terms were combined using the Boolean operator “OR” within each concept group and “AND” between the concept groups. However, the “fear” and “recurrence” concepts were combined using a proximity operator (*adj4*) in most databases to ensure co-occurrence within a 4-word context. The research team designed the search strategy in collaboration with an experienced librarian, and it was adapted for each database. We searched the reference lists of all studies included in the review to identify additional studies.

We searched 5 electronic databases: CINAHL, PsycInfo, the Cochrane Central Register of Controlled Trials (CENTRAL), Embase, and MEDLINE. To capture relevant gray literature, we also searched 2 gray literature sources: ProQuest Dissertations & Theses Global and the World Health Organization International Clinical Trials Registry Platform. These sources were searched from January 1980 to April 2024, given that the concept of FCR was initially raised in the early 1980s [1]. The detailed search strategy and results are provided in Table S1 in Multimedia Appendix 1. We imported the search results from all databases into Covidence [27] and removed duplicates.

### Study Selection

Two authors (NL and TOL) screened titles and abstracts independently for assessment against the inclusion criteria. A third author (CW) resolved any disagreements. A flowchart was created to standardize article selection decisions between screeners (Figure S1 in Multimedia Appendix 1). Two independent reviewers (NL and CW) assessed the full texts in detail against the inclusion criteria. Any disagreements between the reviewers were resolved through discussion with a third author (LR). Reasons for exclusion at the full-text stage were recorded and are presented in the PRISMA-ScR flow diagram (see the Results section).

### Data Extraction

We piloted the data extraction form on the first 5 eligible studies and modified it accordingly. A data dictionary was developed with collective input from the author team to specify all elements of the data extraction form (Table S2 in Multimedia Appendix 1). One reviewer (NL) then extracted the data in Microsoft Excel under the supervision of the research team to ensure adherence to the predefined extraction protocol. The extracted data included:

- General information (author, year of publication, title, journal, country, and study aim);
- Participant characteristics (sample size, age, gender, diagnosis, inclusion criteria, and cancer stage);
- Concept-related information (definition of FCR/progression, study design, intervention timepoint, intervention components, intervention provider, provider qualification, recruitment method, intervention frequency/intervals/duration, definition of control group (if applicable), follow-up timepoints, theoretical framework, outcomes, measurement tool, and result);
- Context (setting and format of delivery);
- Study methodology, key findings, and research gaps.

### Data Analysis and Presentation

Given the heterogeneity across included studies, we conducted a descriptive analysis approach to synthesize the findings. Descriptive numerical summaries were presented through tables and figures to map the characteristics of the included studies, and the findings were synthesized narratively [28]. The synthesis was structured around the review questions.

We summarized the characteristics of the identified studies by reporting frequencies and percentages. We also extracted how FCR was defined and which measurement tools were used in each study and grouped these definitions and measures into tables separately. Key intervention content (ie, components, sample sizes, measurement tools, study design, and reported statistical significance) was summarized. To clearly present the diverse intervention approaches for reducing FCR, we classified the interventions according to their theoretical basis (including blended interventions, cognitive behavioral therapy [CBT], mindfulness-based intervention [MBI], acceptance and commitment therapy [ACT], tailored psychoeducational interventions, and others) with categories developed inductively from the included studies. We created a harvest plot to visually present the distribution of interventions across cancer types and study designs.

## Results

### Overview

**Figure 1.**
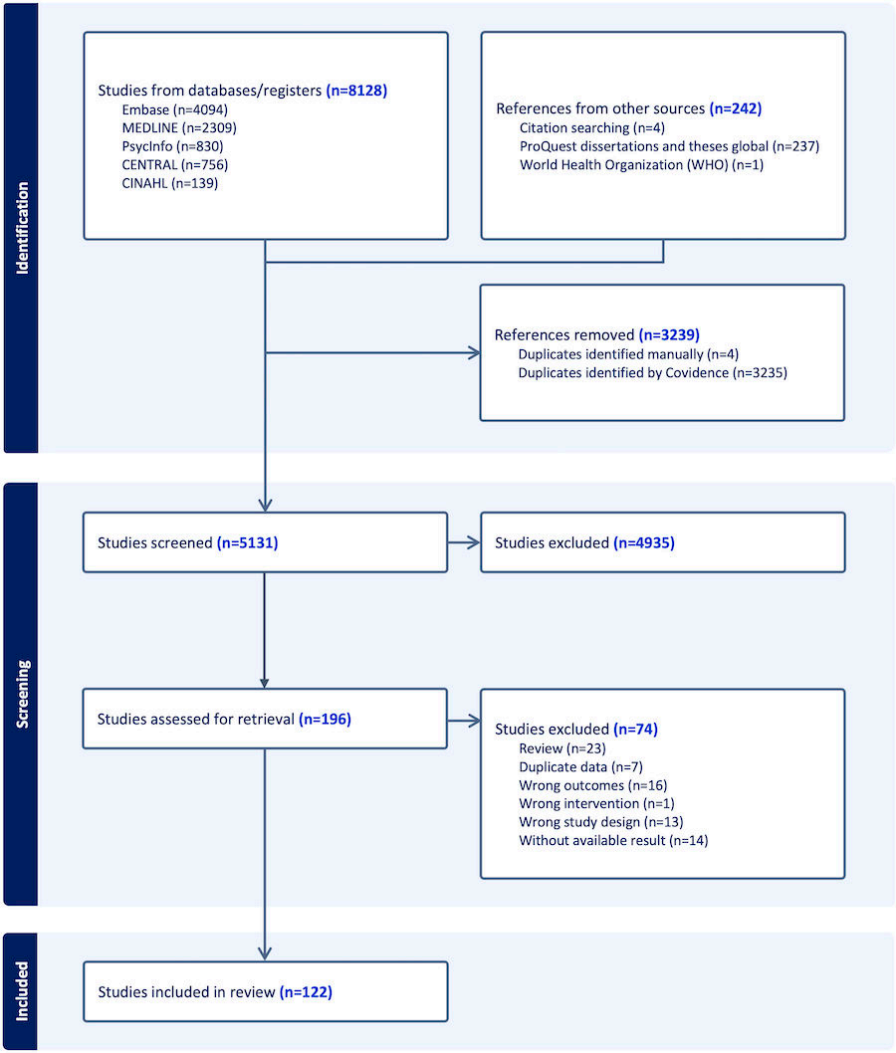
PRISMA-ScR (Preferred Reporting Items for Systematic Reviews and Meta-Analyses extension for Scoping Reviews) flowchart.

We identified 8128 citations through database searches and 242 additional records from gray literature and reference tracking. After removing duplicates, 5131 titles and abstracts were screened, with 4935 excluded. We assessed the full texts of the remaining 196 articles and excluded 74 of them. A total of 122 studies were included in the review (Figure 1).

Sixty-five (53.3%) studies were published within the last 5 years (Table 1). The United States accounted for the highest number of studies (n=35, 28.7%). Forty-eight (39.3%) studies focus on breast cancer, followed by 47 (38.5%) studies that included participants with more than one cancer type. The majority of studies (n=64, 52.5%) were RCTs.

**Table 1.** Characteristics of the included studies (n=122).

Category	Values, n (%)
Publish date	
2009‐2014	14 (11.5)
2015‐2019	43 (35.2)
2020‐2024	65 (53.3)
Geographic region (n=126)^a^	
North America	42 (33.3)
Europe	40 (31.7)
Asia	22 (17.5)
Oceania	20 (15.9)
South America	2 (1.6)
Targeted population	
Breast cancer	48 (39.3)
Gynecologic cancer	8 (6.6)
Melanoma	4 (3.2)
Colorectal cancer	3 (2.5)
Glioma	3 (2.5)
Prostate cancer	2 (1.7)
Renal	2 (1.7)
Other types of cancer	5 (4.0)
Stomach	1 (0.8)
Hypopharyngeal	1 (0.8)
Testicular	1 (0.8)
Oral	1 (0.8)
Lung	1 (0.8)
Any type (more than one type of cancer)	47 (38.5)
Most breast cancer	29 (23.8)
Most hematological cancers	3 (2.5)
Most head and neck cancers	1 (0.8)
Most lung cancer	1 (0.8)
Most lymphoma cancer	1 (0.8)
Mixed	12 (9.8)
Study design	
Randomized controlled trial	64 (52.5)
Pre-post study	44 (36.1)
Mixed methods research	11 (9.0)
Nonrandomized trial study	2 (1.6)
Observational design	1 (0.8)
Intervention (by theoretical base)	
Blended interventions	38 (31.1)
Cognitive behavioral therapy	26 (21.3)
Mindfulness-based interventions	24 (19.7)
Acceptance and commitment therapy	15 (12.3)
Tailored psychoeducational intervention	13 (10.6)
Others	6 (5.0)
Mode of delivery	
In-person	75 (61.5)
Remote (app/web/videoconference/phone call)	34 (27.9)
Mixed (in-person+ remote)	13 (10.6)
Disciplinary specialist (ie, person delivering the intervention)	
Psychologists	68 (55.8)
Other researchers (eg, social workers/graduate students)	20 (16.4)
Any occupation	15 (12.3)
Nurses	7 (5.7)
Doctors	7 (5.7)
Not available	5 (4.1)

a Although 122 studies were included, some were conducted in more than one country; therefore, the total geographic region count is 126.

The interventions were categorized according to their theoretical basis (Table 1). Blended interventions (different combinations of CBT, mindfulness, ACT, and other strategies) formed the largest category (n=38, 31.1%), followed by CBT (n=26, 21.3%) and MBIs (n=24, 19.7%). All studies implemented interventions post diagnosis (Table S3 in Multimedia Appendix 1). In 38 (31.1%) studies, researchers did not report the exact clinical timepoint of intervention delivery. Thirty-six (29.5%) trials included “people who had completed or were still undergoing cancer treatment.” The remaining 48 (39.3%) targeted “people who were disease-free,” with the timing of intervention delivery ranging from 3 months to 15 years following primary cancer treatment. Most interventions were delivered by disciplinary specialists (n=117, 95.9%), with psychologists being the most common providers (n=68, 55.8%).

### The Definition of FCR

Only 28 (23%) studies identified which FCR definition they used. Among them, 9 (32.1%) studies used Vickberg’s definition [28-36], 18 (64.3%) studies used Lebel et al definition [37-54], and 1 (3.6%) study [55] cited the FCR definition by Herschbach and Dinkel [56]. These three definitions are summarized in Table 2. Vickberg’s [57] definition, developed for breast cancer patients, describes FCR as the fear that cancer may return either in the same location or in another part of the body. Lebel et al [2] proposed a definition based on a Delphi consensus process, defining FCR as “fear, worry, or concern relating to the possibility that cancer will come back or progress.” Herschbach and Dinkel [56] introduced the concept of fear of progression (FoP), which specifically highlights concerns about disease progression and its associated psychosocial consequences.

**Table 2.** Definition of FCR^a^.

Author	Definition
Vickberg (2003) [57]	“Fear or concern about the return or progression of the disease in the same organ or other parts of the body.”
Lebel et al (2016) [2]	“Fear, worry, or concern relating to the possibility that cancer will come back or progress.”
Herschbach and Dinkel (2014) [56]	“Fear of progression (FoP) is defined as the fear and anxiety of patients whose disease will progress or recur with all its social and biological consequences.”

a FCR: fear of cancer recurrence.

### FCR Measurement Tools

Eighteen validated measurement tools were identified (Table S4 in Multimedia Appendix 1). Thirteen (72.2%) were specifically designed to assess FCR/FoP, while the remaining 5 (27.8%) included FCR-related items as part of broader assessments. The Fear of Cancer Recurrence Inventory [58] was the most frequently used tool, appearing in 40 (31.7%) studies. Other commonly used tools included the Concerns About Recurrence Scale [57], used in 20 (15.9%) studies, and the Fear of Cancer Recurrence Inventory-Short Form [59], used in 18 (14.3%) studies.

### Intervention Characteristics and Effects

#### Summary

**Figure 2.**
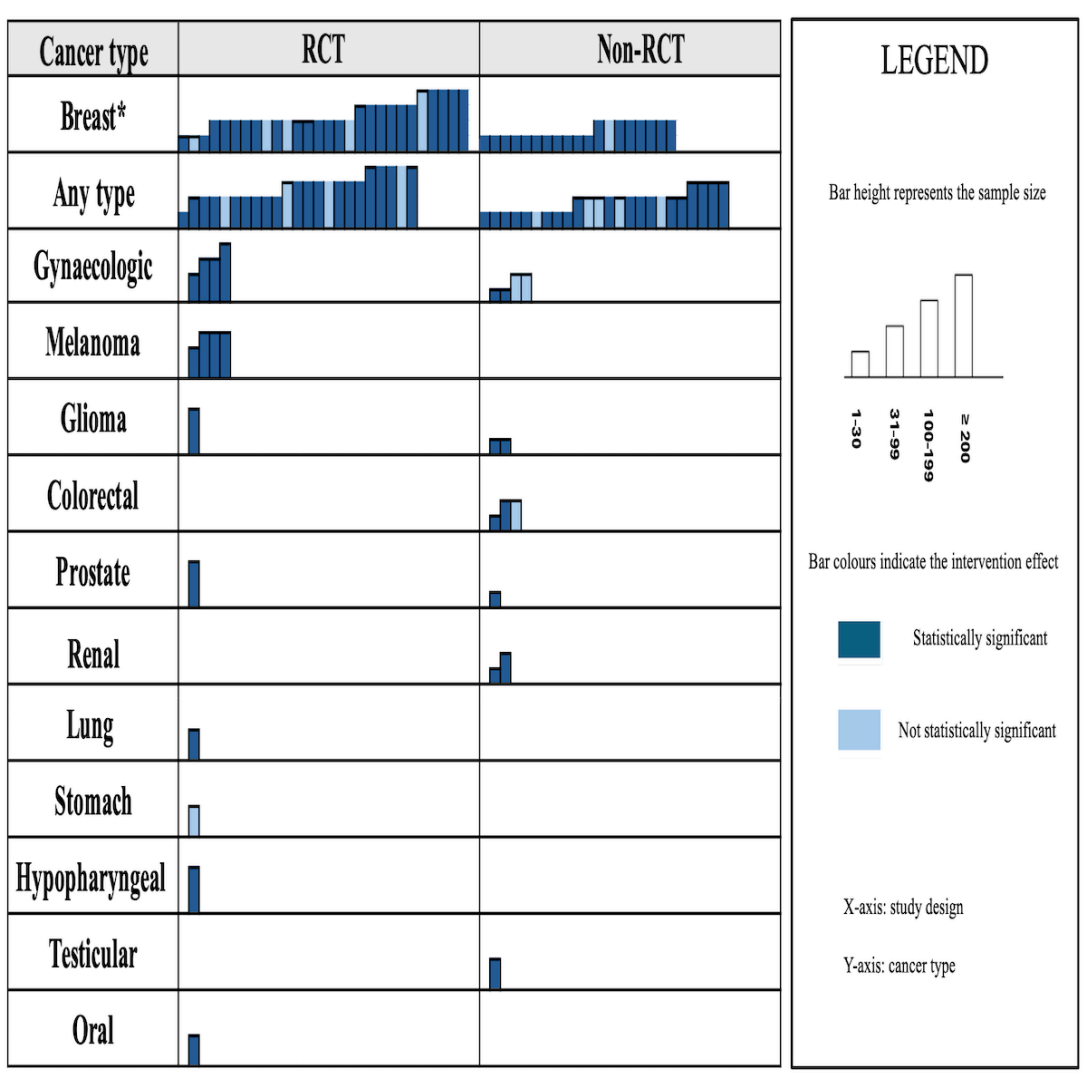
Harvest plot showing the distribution of interventions by study design and cancer type. Each bar represents one study. Bar colors indicate the intervention effect, and bar height reflects the sample size. “Any type” refers to studies that include more than one type of cancer. Blue bars indicate a statistically significant effect (*P*<.05) in reducing fear of cancer recurrence; light blue bars indicate no statistically significant effect. Studies are grouped by cancer type (y-axis) and study design (randomized controlled trial [RCT] vs non-RCT; x-axis). * represents one study with missing sample size data. Within each group, studies are ordered from left to right by sample size, starting with the smallest sample size on the left.

We summarized author, year, intervention, sample size, measurement tools, statistical significance, and study design (Table 3). The distribution and reported effects of interventions across cancer types and study designs are summarized visually (Figure 2). Detailed characteristics of statistically significant interventions are outlined (Table S5 in Multimedia Appendix 1).

**Table 3.** The intervention and effect.

Authors (year)	Sample, n	Intervention	FCR tools^a^	Statistically significant^b^	Research design
AhmadiQaragezlou et al 2020 [29]	38	Mindfulness-based stress reduction (MBSR) program	FCRI	Yes	RCT^c^
Akkol-Solakoglu and Hevey 2023 [60]	72	Internet-delivered cognitive behavioral therapy (CBT)	CWS	No	RCT
Akechi et al 2021 [61]	59	Collaborative care intervention—problem-solving treatment (PST) and behavioral activation program (BA) (modified version)	CARS	No	RCT
Akechi et al 2023 [62]	444	Smartphone-based PST (Kaiketsu-App) and behavioral activation program (Genki-App)—problem-solving treatment (PST) and behavioral activation program (BA) (modified version)	CARS and FCRI-SF	Yes	RCT
Almeida et al 2022 [37]	17	Emotion-focused therapy (EFT)—humanistic-experiential psychotherapy stemming from person-centered, gestalt, and existential traditions	PQ-FCR	Yes	Pre-post study
Ananeva 2020 [63]	N/A^d^	ConquerFear (CF)—Common-Sense Model (CSM) of illness, the Self-Regulatory Executive Function model (S-REF; targets metacognitions), and Relational Frame Theory (RFT; theoretical basis for acceptance and commitment therapy)	FCRI	Yes	Pre-post study
Arch and Mitchell 2016 [64]	42	Valued Living (VL) intervention	CARS	Yes	Pre-post study
Arch et al 2021 [65]	134	Valued Living (VL) intervention	CARS	Yes	RCT
Arch et al 2024 [66]	29	Written exposure-based coping intervention (EASE)	CARS and FOP-Q-SF	Yes	Pre-post study
Beith et al 2017 [67]	222	ConquerFear (CF)—Common-Sense Model (CSM) of illness, the Self-Regulatory Executive Function model (S-REF; targets metacognitions), and Relational Frame Theory (RFT; theoretical basis for acceptance and commitment therapy)	FCRI	Yes	RCT
Bergerot et al 2022 [68]	23	Smartphone app–based mindfulness program	FCR-7	Yes	Pre-post study
Bergerot et al 2023 [69]	41	Mindfulness-Based Cancer Survivorship Journey	FCR-7	Yes	Pre-post study
Bin et al 2023 [70]	98	Observation group based on the Rosenthal effect	FCRI	No	RCT
Brooker et al 2020 [71]	173	Mindful Self-Compassion (MSC) program	FCRI-SF	Yes	Pre-post study
Burm et al 2019 [38]	88	Blended cognitive behavioral therapy (CBT)	CWS	Yes	RCT
Butow et al 2017 [72]	222	ConquerFear (CF)—Common-Sense Model (CSM) of illness, the Self-Regulatory Executive Function model (S-REF; targets metacognitions), and Relational Frame Theory (RFT; theoretical basis for acceptance and commitment therapy)	FCRI	Yes	RCT
Chambers et al 2012 [73]	19	Mindfulness-based cognitive therapy group intervention (MBCT)	MAX-PC	Yes	Mixed methods research
Chang 2025 [74]	50	Integrated Mindfulness-Based Fitness Training (MBFT) program	ASC	Yes	RCT
Cheng et al 2021 [75]	172	Mindfulness-Based Fitness Training (MBFT) program	FCRS	Yes	RCT
Cillessen et al 2018 [76]	245	Group face-to-face and individual internet-based mindfulness-based cognitive therapy (MBCT and eMBCT)	FCRI-SF	No	RCT
Cohen et al 2022 [31]	19	Mindfulness-based cognitive therapy (MBCT)	FCRI	Yes	Pre-post study
Compen et al 2018 [77]	245	Face-to-face and internet-based mindfulness-based cognitive therapy (MBCT and eMBCT)	FCRI	Yes	RCT
Crane-Okada et al 2012 [78]	49	Mindful Movement Program (MMP)	FCRS	Yes	RCT
Davidson et al 2018 [30]	16	Mini‐Adjustment to Fear, Threat, and Expectation of Recurrence (Mini‐AFTERc) intervention	FCRI-SF	Yes	Pre-post study
Deuning-Smit et al 2024 [39]	81	Cognitive behavioral therapy (CBT)	CWS-6 and FCRI-SF	Yes	Mixed methods research
Dieng et al 2016 [79]	164	Melanoma care program	FCRI	Yes	RCT
Dieng et al 2019 [80]	164	Melanoma care program	FCRI	Yes	RCT
Dieng et al 2020 [81]	164	Melanoma care program	FCRI	Yes	RCT
Dirkse et al 2020 [82]	86	Internet-delivered cognitive behavioral therapy	FCRI-SF	Yes	RCT
Dodds et al 2015 [83]	33	Cognitively Based Compassion Training (CBCT) intervention	FCRI	Yes	RCT
Döking et al 2021 [84]	1	Combined face-to-face and online cognitive behavioral therapy	CWS	Yes	Mixed methods research
Eckert et al 2020 [85]	15	Mindfulness-enhanced cognitive stress management (MECSM) group intervention	CWS	No	Pre-post study
Fishbein et al 2023 [86]	113	Valued Living (VL) intervention	CARS	No	RCT
Fishbein et al 2022 [87]	134	Valued Living (VL) intervention	CARS	Yes	RCT
Fishbein and Arch 2022 [88]	73	Valued Living (VL) intervention	CARS	No	RCT
Fisher et al 2019 [89]	27	Metacognitive therapy (MCT)—trans-diagnostic theory of psychopathology, the Self-Regulatory Executive Function (S-REF) mode	FCRI	No	Pre-post study
Fisher et al 2017 [90]	4	Metacognitive therapy (MCT)—trans-diagnostic theory of psychopathology, the Self-Regulatory Executive Function (S-REF) mode	FCRI	Yes	Nonconcurrent multiple baseline design
Frangou et al 2021 [91]	182	Psychological support intervention primarily incorporated cognitive behavioral therapy (CBT), Mindfulness and acceptance and commitment therapy (ACT) elements	FOP-Q-SF	Yes	RCT
Gonzalez-Hernandez et al 2018 [92]	56	Cognitively-Based Compassion Training (CBCT)	FCRI	Yes	RCT
Hall et al 2022 [93]	23	A mind-body resiliency intervention—relaxation response (RR), cognitive behavioral based	FCRI-SF	Yes	Mixed methods research
Hall et al 2020 [94]	23	A mind-body resiliency intervention—relaxation response (RR), cognitive behavioral based	FCRI	Yes	Pre-post study
Hasannezhad Reskati et al 2020 [55]	30	Acceptance and commitment therapy (ACT)	FOP-Q	Yes	RCT
Herschbach 2012 [95]	174	Cognitive behavioral group therapy (CBT)	FOP-Q	Yes	RCT
Herschbach et al 2010 [95]	265	Group Psychotherapy CBT intervention	FOP-Q	Yes	RCT
Howells et al 2019 [91]	107	Cognitive behavioral therapy (CBT)	FOP-Q-SF	Yes	RCT
Humphris and Rogers 2012 [96]	90	AFTER (Adjustment to the Fear, Expectation or Threat of Recurrence) intervention	WOC	Yes	RCT
Imai et al 2019 [97]	38	Problem-solving therapy (PST)	CARS	Yes	Pre-post study
Johns et al 2020 [98]	91	Acceptance and commitment therapy (ACT)	FCRI-SF	Yes	RCT
Kacel 2019 [99]	32	Mindfulness yoga program	FCRI	No	Pre-post study
Lebel et al 2014 [32]	56	Cognitive-existential (CE) group intervention-Leventhal’s common sense model, Mishel’s uncertainty in illness theory and cognitive models of worry	FCRS	Yes	Pre-post study
Lee et al 2022 [100]	54	Mindfulness stress management (MSM) intervention	FCRI	Yes	RCT
Lengacher et al 2016 [101]	322	Mindful Movement Program (MMP)	CARS	Yes	RCT
Lengacher et al 2021 [102]	322	Mindfulness-based stress reduction (MBSR) intervention	CARS	Yes	RCT
Lengacher et al 2009 [103]	84	Mindfulness-based stress reduction (MBSR) intervention	CARS	Yes	RCT
Lengacher et al 2018 [104]	15	Mobile mindfulness-based stress reduction (mMBSR) intervention	CARS	Yes	Pre-post study
Lichtenthal et al 2017 [105]	110	Attention and Interpretation Modification for Fear of Breast Cancer Recurrence (AIM-FBCR)	CARS	Yes	RCT
Li et al 2021 [106]	140	Psychological nursing intervention	FoP-Q-SF	Yes	RCT
Liu et al 2021 [107]	61	Clinician Intervention to address Fear of Cancer Recurrence (CIFeR)—FCR theoretical models (self-regulation model of illness, self-regulatory executive functioning model, family-based model, uncertainty in illness theory, social-cognitive processing theory, and terror management theory)	FCRI	Yes	Pre-post study
Loughan et al 2022 [108]	12	Managing Cancer and Living Meaningfully (CALM) intervention	FCR-7	No	Pre-post study
Loughan et al 2021 [109]	10	Managing Cancer and Living Meaningfully (CALM) intervention	FCR-7	No	Mixed methods research
Luberto et al 2019 [110]	1	Mindfulness-based cognitive therapy (MBCT)	CWS	Yes	Mixed methods research
Luigjes‐Huizer et al 2023 [40]	167	Online primary care intervention—cognitive behavioral therapy and the model on FCR by Lee‐Jones	FCRI-SF	Yes	RCT
Maheu et al 2023 [111]	164	Fear of cancer recurrence therapy (FORT) based on cognitive existential (CE) approach, which combines elements of modern existential therapies and cognitive behavioral therapy (CBT)	FCRI	Yes	RCT
Manne et al 2017 [112]	352	Communication enhancing intervention (CCI)/supportive counseling (SC)—cognitive-affective social processing theory/encouraging emotional expression, supporting existing coping behaviors, and enhancing self-esteem and autonomy	CARS	Yes	RCT
Martin et al 2020 [113]	114	iHOPE (Help to Overcome Problems Effectively) intervention	QLACS	Yes	Pre-post study
McHale et al 2024 [41]	92	Mini‐Adjustment to Fear, Threat, and Expectation of Recurrence (Mini‐AFTERc) intervention	FCR-4	Yes	Nonrandomized trial study
Merckaert et al 2017 [114]	170	Multiple‐component structured manualized group intervention (MGI)—cognitive behavioral and hypnosis components	FCRI	Yes	RCT
Momino et al 2017 [115]	40	Collaborative care intervention—problem-solving therapy (PST) and behavioral activation therapy (BAT)	CARS	No	Pre-post study
Montesinos and Francisco 2016 [116]	12	Abridged version of acceptance and commitment therapy (ACT)	Mini-MAC	Yes	Pre-post study
Murphy et al 2020 [117]	114	Internet‐delivered cognitive behavioral therapy (iCBT)	FCRI	Yes	RCT
Neubert et al 2023 [118]	157	Video Sequence-Based Intervention	FoP-Q-SF	No	RCT
Nguyen et al 2022 [119]	57	Virtual telephone coaching program	FCRI	Yes	Pre-post study
Otto 2015 [120]	67	Gratitude intervention	CARS	Yes	Pre-post study
Park et al 2020 [121]	74	Mindfulness-based cognitive therapy (MBCT) program	CARS	Yes	RCT
Peng et al 2022 [33]	65	Mindfulness-based stress reduction (MBSR) intervention	FCRI-SF	Yes	RCT
Pradhan et al 2021 [122]	62	Online Fear of Cancer Recurrence Booklet—adapted from the ConquerFear (Common Sense Model of Illness, Self-Regulatory Executive Function Model (S-REF), and Relational Frame Theory)	FOP-Q-SF	No	Pre-post study
Reb et al 2020 [43]	31	ConquerFear (CF)—contemporary cognitive-processing model from Metacognitive Therapy (MCT) and acceptance and commitment therapy (ACT) principles	FOP-Q-SF	Yes	Pre-post study
Reb et al 2020 [44]	2	Day-by-day (DBD) intervention—ConquerFear based (Common Sense Model of Illness, Self-Regulatory Executive Function Model (S-REF), and Relational Frame Theory)	FOP-Q-SF	Yes	Pre-post study
Smith et al 2020 [123]	44	Online psychological self-management intervention—cognitive behavioral, acceptance and commitment, metacognitive, and relaxation therapy	FCRI-SF	Yes	Mixed methods research
Rudolph et al 2018 [124]	10	Acceptance and commitment therapy (ACT)	FoP-Q	Yes	Pre-post study
Russell et al 2019 [125]	69	Online mindfulness-based program (MBIs)	FCRI	Yes	RCT
Sajadian et al 2021[126]	30	Psycho-spiritual therapy	FCRI	Yes	RCT
Saltbæk et al 2024 [127]	503	Nurse-led individualized follow-up	CARQ	Yes	RCT
Salazar-Alejo et al 2023 [128]	97	Online mindfulness-based stress reduction (MBSR) intervention	CWS	Yes	RCT
Sarizadeh et al 2018 [129]	20	Acceptance and commitment therapy (ACT)	FCRI	Yes	Pre-post study
Sauer and Maatouk 2021 [130]	30	Acceptance and commitment therapy (ACT)	N/A	Yes	Pre-post study
Savard et al 2018 [45]	38	Group cognitive behavioral therapy (CBT)	FCRI	Yes	Mixed methods research
Sakai et al 2024 [131]	1	Acceptance and commitment therapy (ACT)	CARS	Yes	Pre-post study
Schlecht et al 2023 [132]	155	Video-based intervention—ACT, mindfulness-based	FOP-Q-SF	Yes	Observational design
Sharpe et al 2019 [46]	152	ConquerFear (CF)—a combination of metacognitive therapy, the self-regulation theory, and acceptance and commitment therapy	FCRI	Yes	Pre-post study
Shih et al 2014 [133]	96	ConquerFear (CF)—Self-Regulatory Executive Function Model and Relational Frame Theory	FCRI	Yes	RCT
Shumay et al 2013 [134]	28	Acceptance and commitment therapy (ACT)	FCRI	No	RCT
Sinclair et al 2023 [135]	97	Acceptance and commitment therapy (ACT)	FCRI-SF	Yes	Pre-post study
Sinclair et al 2020 [136]	79	Acceptance and commitment therapy (ACT) and patient education intervention (adapted from ConquerFear intervention)—Common Sense Model of Illness, Self-Regulatory Executive Function Model (S-REF), and Relational Frame Theory	FACT	Yes	Mixed methods research
Smith et al 2024 [47]	54	ConquerFear (CF)—Common Sense Model of Illness, Self-Regulatory Executive Function Model (S-REF), and Relational Frame Theory	FCRI-SF	Yes	Mixed methods research
Smith et al 2015 [34]	8	ConquerFear (CF)—Common Sense Model of Illness, Self-Regulatory Executive Function Model (S-REF), and Relational Frame Theory	FCRI	Yes	Pre-post study
Steinecke et al 2022 [137]	33	Psychobiological group therapy program—behavioral strategies as well as mindfulness	N/A	Yes	Pre-post study
Subnis 2014 [138]	40	Online expressive writing intervention (EW)	FCRI-SF	Yes	RCT
Sun et al 2023 [139]	40	Traditional Chinese Medicine (TCM) interventions combined group psychotherapy, mindfulness, and cognitive behavioral therapy-based	FCRI	Yes	Pre-post study
Tauber et al 2023 [48]	85	ConquerFear (CF)—Common Sense Model of Illness, Self-Regulatory Executive Function Model (S-REF), and Relational Frame Theory	FCRI	Yes	RCT
Tauber et al 2022 [49]	27	ConquerFear (CF)—Common Sense Model of Illness, Self-Regulatory Executive Function Model (S-REF), and Relational Frame Theory	FCRI-SF	Yes	RCT
Thewes et al 2012 [140]	8	ConquerFear (CF)—Common Sense Model of Illness, Self-Regulatory Executive Function Model (S-REF), and Relational Frame Theory	FCRI-SF	Yes	Pre-post study
Tomei et al 2018 [50]	25	Cognitive-existential (CE) psychotherapy intervention—Leventhal’s common sense model, Mishel’s uncertainty in illness, and cognitive models of worry	FCRI	Yes	RCT
Tomei et al 2016 [35]	3	Cognitive-existential (CE) psychotherapy intervention—Leventhal’s common sense model, Mishel’s uncertainty in illness, and cognitive models of worry	FCRI	Yes	Pre-post study
Tomei et al 2014 [141]	1	Cognitive-existential (CE) psychotherapy intervention—Leventhal’s common sense model, Mishel’s uncertainty in illness, and cognitive models of worry	FCRI	No	Pre-post study
Tran 2025 [142]	53	Fear-Less self-management intervention (stepped-care framework)	FCRI-SF	Yes	Pre-post study
Ulfig 2020 [36]	35	Cognitive behavioral therapy (CBT)	FACT	Yes	RCT
Ulfig 2020 [36]	115	Cognitive behavioral therapy (CBT)	FACT	Yes	Pre-post study
Valcu et al 2023 [143]	9	Cognitive behavioral therapy (CBT)	FoP-Q-SF	Yes	Pre-post study
van de Wal et al 2018 [51]	1	Cognitive behavioral therapy (CBT)	CWS and FCRI	Yes	Mixed methods research
van de Wal et al 2015 [144]	1	Blended cognitive behavioral therapy (CBT)	CWS	Yes	Pre-post study
van de Wal et al 2017 [145]	88	Blended cognitive behavioral therapy (CBT)	CWS	Yes	RCT
van Helmondt et al 2023 [52]	262	Cognitive behavioral therapy (CBT)	FCRI	No	RCT
Victorson et al 2012 [146]	115	Mindfulness-based stress reduction intervention (MBCT)	MAX-PC	Yes	RCT
Wagner et al 2021 [147]	196	Cognitive behavioral therapy (CBT)	FCRI	Yes	RCT
Wang et al 2023 [53]	103	Managing cancer and living meaningfully (CALM)	CWS	Yes	RCT
Wang et al 2023 [148]	98	Meaning-centered group psychotherapy (MCGP)—Viktor Frankl’s theory and principles, and contains some existential elements	FOP-Q-SF	Yes	RCT
Weis et al 2020 [149]	50	Psychoeducational (PE) group intervention	FoP-Q-SF	Yes	Nonrandomized trial study
Yoon et al 2023 [150]	41	Interactive coaching intervention	FCRI-SF	No	Pre-post study
Zhang et al 2022 [151]	98	Managing cancer and living meaningfully (CALM)	CARS	No	RCT
Zhao et al 2023 [54]	80	Managing cancer and living meaningfully (CALM) intervention	FCRI	Yes	RCT
Zhao and Xu 2021 [152]	258	Comprehensive nursing based on cognitive behavioral intervention	FoP-Q-SF	Yes	RCT

a Fear of cancer recurrence (FCR) tools used are as follows—ASC: Assessment of Survivor Concerns; CARQ: Concerns About Recurrence Questionnaire; CARS: Concerns About Recurrence Scale; CWS: Cancer Worry Scale; CWS-6: 6-Item Cancer Worry Scale; FACT: Functional Assessment of Cancer Therapy; FCR-4: Fears of Cancer Recurrence, 4-Item Version; FCR-7: Fears of Cancer Recurrence, 7-Item Version; FCRI: Fear of Cancer Recurrence Inventory; FCRI-SF: Fear of Cancer Recurrence Inventory—Short Form; FCRS: Fear of Cancer Recurrence Scale; FoP-Q: Fear of Progression Questionnaire; FOP-Q-SF: Short-Form Fear of Progression Scale; MAX-PC: Memorial Anxiety Scale for Prostate Cancer; Mini-MAC: Mini-Mental Adjustment to Cancer Scale; PQ-FCR: Personal Questionnaire—Fear of Recurrence; QLACS: Quality of Life in Adult Cancer Survivors Scale; WOC: Worry of Cancer Scale.

b “Yes” means that the intervention had a statistically significant mitigating effect on fear of cancer recurrence (FCR); “No” means that the intervention did not have a statistically significant mitigating effect on FCR.

c RCT: randomized controlled trial.

d N/A: not available.

#### Blended Interventions

Thirty-eight (31.1%) studies evaluated the effect of blended interventions [31,32,34,35,37,40,42-44,46-50,53,61-63,67,72,89-91,93,94,107,111,112,114,115,123,132,133,136,137,139-141]. Most of them (n=23, 60.5%) combined CBT with other interventions (eg, mindfulness, ACT, and other strategies). Eighteen (47.4%) studies used a pre-post study design [31,32,34,35,37,42-44,46,63,89,94,107,115,137,139-141]. Fourteen (36.8%) studies were RCTs [40,48-50,53,61,62,67,72,91,111,112,133,153].

The majority of studies (n=33, 86.8%) reported statistically significant effects. Among them, 22 (66.7%) were conducted in person by disciplinary specialists [31,32,34,37,46,48,49,63,67,72,90,91,93,94,107,111,114,133,136,137,140,148], 8 (24.2%) were delivered remotely [40,43,44,47,62,123,132,139], and 3 (9.1%) used a hybrid format, combining both in-person and remote delivery [35,50,112]. These interventions typically followed a weekly format, lasting from 5 to 12 weeks. Individual sessions ranged from 10 minutes to 2 hours. Moreover, the length of the follow-up period varied, with assessments conducted from immediately after the intervention up to 2 years.

#### CBT

Twenty-six (21.3%) studies evaluated the effect of CBT [30,36,38,39,41,45,51,52,60,82-84,91,92,95,96,105,119,143-145,147,152,154-156]. The majority of the studies (n=16, 61.5%) were RCTs [36,38,52,60,82,83,91,92,95,96,105,117,145,147,152,155], followed by 5 (19.2%) pre-post studies [30,119,143,144,154], 4 (15.4%) mixed methods studies [39,45,51,84], and 1 (3.9%) non-RCT [41].

Twenty-three (88.5%) studies reported statistically significant effects. Among them, 11 (47.8%) were delivered face to face by disciplinary specialists [36,39,45,83,95,96,105,152,154,155,157], while 7 (30.4%) studies used remote delivery methods [30,41,82,117,119,143,147], followed by 5 (21.8%) delivered both in-person and online [38,51,84,144,145]. These interventions typically followed a weekly format, lasting from 1 to 12 weeks. Individual sessions ranged from 10 minutes to 2.5 hours, with follow-up periods spanning from immediately after the intervention to 15 months.

#### Mindfulness-Based Interventions

Twenty-four (19.7%) studies evaluated the effect of MBIs [29,33,68,69,71,73-78,85,99-104,110,121,125,126,128,146]. Seven (29.2%) studies [29,33,100,102-104,128] used mindfulness-based stress reduction interventions. Six (25%) [73,76,77,110,121,146] applied mindfulness-based cognitive therapy. Four (16.7%) studies [74,75,78,101] evaluated mindfulness-based fitness or movement programs, and 1 (4.2%) study [71] conducted a mindful self-compassion program. Additionally, 6 (25%) studies [68,69,85,99,125,126] incorporated a mix of mindfulness components. Most studies (n=16, 66.7%) were RCTs [29,33,74-78,100-103,121,125,126,128,146], followed by 6 (25%) pre-post studies [68,69,71,85,99,104] and 2 (8.3%) mixed methods studies [73,110].

The majority (n=21, 87.5%) reported statistically significant effects. Disciplinary specialists delivered most effective interventions (n=14, 66.7%) face-to-face, while 7 (33.3%) studies delivered interventions remotely via websites [74,104,125,128], and apps [62,68,69]. The majority of interventions (n=19, 90.5%) involved weekly sessions lasting between 5 and 12 weeks, with each session lasting between 90 minutes and 2.5 hours. All studies measured outcomes immediately post intervention, and the longest follow-up lasted 1 year.

#### Acceptance and Commitment Therapy

Fifteen (12.3%) studies evaluated the effect of ACT [55,64,65,86-88,98,116,118,124,129-131,134,135]. Among them, 8 (53.3%) studies were RCTs [55,65,86-88,98,118,134], while 7 (46.7%) followed a pre-post design [64,116,124,129-131,135].

Most studies (n=11, 73.3%) reported statistically significant effects. All effective interventions were delivered in person by disciplinary specialists, following a weekly format that ranged from a single session to 8 weeks. Each session lasted between 50 and 120 minutes. Follow-up periods varied widely, from immediately post intervention to up to 8 months.

#### Tailored Psychoeducational Interventions

Thirteen (10.6%) trials evaluated the effect of tailored psychoeducational interventions. Among them, 5 (38.5%) studies [53,54,108,109,151] evaluated the effect of an intervention named the Managing Cancer and Living Meaningfully intervention. Three (23.1%) studies evaluated the effect of a melanoma care program [79-81]. The majority of studies were RCTs (n=9, 69.2%) [53,54,70,79-81,106,127,151].

Most studies (n=9, 69.2%) reported statistically significant effects. All interventions were delivered by disciplinary specialists, with 8 (88.9%) delivered face-to-face. The duration of effective interventions ranged from 4 to 24 weeks, with individual sessions lasting between 30 minutes and 4 hours. Follow-up periods ranged from immediately post intervention to up to 3 years.

#### Others

In addition to the widely used interventions described above, 6 (4.9%) studies explored alternative approaches, including writing interventions [66,138], a holistic biopsychosocial intervention [150], a problem-solving intervention [97], an intervention grounded in hope theories [113], and an intervention inspired by broadening and building theories [158]. Among them, 5 (83.3%) studies used a pre-post design [66,97,113,120,150], and 1 used an RCT design [138]. Five (83.3%) reported statistically significant effects and were delivered online by disciplinary specialists. Follow-up periods ranged from immediately post intervention to 4.5 months.

## Discussion

### Principal Findings

This scoping review is the first to map all psychological intervention strategies for alleviating FCR across cancer types and to identify key gaps in existing evidence. From our review, only about one-fifth of the studies reported the definition of FCR used. Eighteen validated FCR measurement tools were identified. Blended interventions, CBT, and MBIs were the most common approaches, typically delivered face-to-face by disciplinary specialists. Breast cancer populations dominated the evidence base, while other cancer types received limited attention. Most interventions reported statistically significant effects in reducing FCR, although the wide variation in study designs, intervention components, outcome measures, and follow-up durations makes meaningful interpretation difficult.

A clear definition of FCR is critically important, as it underpins the identification of its clinical manifestations, supports accurate diagnosis in clinical settings, and informs the design of targeted interventions that address the specific fears and cognitive patterns associated with cancer recurrence [21,32]. Much of the existing literature treats the definitions of FCR and FoP interchangeably [159,160]. However, Coutts-Bain et al [161] conducted a factor analysis and found that, although FCR and FoP are related, they are structurally distinct psychological constructs. It challenges the previous assumption that the two are the same construct and emphasizes the importance of clearly distinguishing between FCR and FoP in clinical practice and research. Conceptually, FCR is more relevant to cancer survivors who have completed curative treatment and are in remission, encompassing concerns about the cancer returning after a period of being disease-free. In contrast, individuals with metastatic cancer are less likely to fear recurrence and more likely to experience fear of progression, worrying about the cancer worsening or spreading further due to the ongoing nature of their illness [162]. Consequently, clearly distinguishing between FCR and FoP in clinical practice and research not only facilitates the development of more effective, targeted interventions but also enhances the accuracy of research findings and enables the provision of more tailored psychological support to cancer survivors.

Blended interventions were the most commonly investigated for reducing FCR among cancer survivors, with numerous studies reporting positive outcomes. This finding aligns with another review of 13 studies [163], which also identified that interventions combining CBT and MBI strategies were effective in reducing FCR. In recent years, researchers have proposed that traditional CBT may have limitations in managing FCR, particularly among survivors living with ongoing uncertainty [21,164]. An overemphasis on challenging “irrational” thoughts may leave some people feeling misunderstood or emotionally invalidated, especially when their fears are grounded in real risk rather than cognitive distortion [165]. However, evidence supporting these concerns remains limited. Some scholars regard ACT as a contextual extension of CBT, incorporating core mindfulness-based practices to enhance individuals’ awareness and acceptance of their emotions [166]. Similarly, mindfulness techniques are increasingly integrated into traditional CBT. A prime example of this integration is mindfulness-based cognitive therapy, which combines the cognitive restructuring techniques of CBT with mindfulness practices to help individuals break the cycle of negative thinking and reduce the FCR [121].

The diversity in FCR interventions reflects the lack of a universally agreed theoretical foundation and the variety of disciplinary approaches to understanding cancer-related psychological experiences, which creates challenges for their application in clinical practice. Despite growing research on FCR interventions, cancer survivors still rarely receive support for managing FCR outside the research environment [167]. It is therefore important to identify the core effective components across FCR interventions and to examine whether tailored approaches for specific cancer populations offer advantages over more general interventions [168]. Furthermore, for widespread adoption, considerations such as cost-effectiveness and resource requirements will be essential to ensure the feasibility of dissemination [169,170].

The majority of existing FCR interventions were resource-intensive, requiring in-person delivery over multiple sessions by trained specialists. These findings are also supported by a previous review by Pradhan et al [122]. As FCR is a persistent and commonly unmet concern across cancer survivor populations, there is a pressing need for scalable solutions [47,164]. In addition, a recently published consensus statement [165] indicates that approximately 80% of cancer survivors do not require high-intensity therapy, and that lower-intensity approaches, such as self-guided online programs, psychoeducational resources, or brief interventions delivered via SMS text messages or telephone, can reduce FCR [165]. In this context, mobile health interventions offer greater scalability and accessibility with reduced demands on health care resources, making them a promising alternative [171]; text messaging–based programs have shown positive outcomes in the management of mental health disorders [172] and in improving self-management of chronic conditions [173]. However, there remains a lack of mobile health programs in managing FCR.

In this review, the majority of studies (85%) reported statistically significant effects of interventions on FCR. This trend is consistent with evidence from previous systematic reviews and meta-analyses, which also demonstrated small-to-moderate reductions in FCR across specific psychological interventions [164,174]. However, this predominance of positive findings may be partially explained by publication bias, whereby studies with null or negative results are less likely to be published [175]. Researchers and journals often favor significant or “positive” outcomes, which can distort the evidence base and lead to an overestimation of intervention effectiveness [176]. Selective outcome reporting may further contribute to this bias, as authors might emphasize significant results while downplaying or omitting nonsignificant findings [177]. Small sample sizes in some studies may increase the risk of false positives and inflated effect sizes [178]. Collectively, these factors could mislead clinical decision-making and contribute to the adoption of interventions with uncertain or limited effects. In addition, while most interventions reduced FCR, these findings should be interpreted with caution due to the heterogeneity across the included studies. The interventions varied widely in terms of dose, delivery format, sample size, measurement time points, and study design, all of which pose significant challenges for synthesizing the findings. Such variability limits the comparability of studies and makes it difficult to have a specific conclusion regarding the overall effect of interventions for managing FCR. These challenges are inherent to scoping reviews, which aim to map diverse and broad evidence [28,179]. It is necessary to conduct a systematic review with high-quality RCTs to evaluate the effectiveness of different interventions in the future.

Although FCR was recognized as a clinical concern in the 1980s [1], our review found that interventions only began to appear from around 2009. FCR was underrecognized as a distinct psychological construct and was often assessed as part of broader measures of distress, anxiety, or quality of life previously [7]. The lack of validated tools specifically designed to assess FCR also limited early research and hindered the earlier evaluation of intervention outcomes [58]. Over the past two decades, the growing number of cancer survivors has shifted the focus of cancer care from survival alone to long-term psychological well-being, highlighting the need for FCR-specific interventions [2].

### Limitations

First, this review used a vote-counting approach to summarize intervention effects because of the heterogeneity across studies. However, this means all studies were weighted equally, regardless of their quality, sample size, or effect size, which limits our ability to compare effect estimates or assess the clinical meaningfulness of intervention outcomes. In addition, we did not conduct a quality appraisal of the included studies, consistent with the methodological nature of a scoping review. Future research would benefit from high-quality systematic reviews and meta-analyses that incorporate rigorous risk-of-bias assessments and enable quantitative comparisons of the effectiveness of different intervention modalities.

Second, this review was limited to English-language articles or English-language abstracts, and the exclusion of non-English publications may have reduced the completeness and representativeness of the evidence. This restriction may introduce language bias and underrepresent studies conducted in non-English-speaking regions. Nonetheless, our review included studies from 5 continents, indicating some geographic diversity despite the language limitation. Future reviews should consider removing language restrictions to ensure broader inclusion.

Third, this review relied solely on published information and did not seek additional details from study authors, which may have resulted in incomplete reporting of methodological or contextual elements, such as the specific definitions of FCR used. Future studies would benefit from more detailed reporting and from contacting authors when necessary to obtain missing information.

### Conclusion and Implications

Our scoping review mapped the full range of psychological intervention strategies aimed at alleviating FCR across cancer types. We identified 122 studies encompassing diverse FCR interventions. FCR has been the focus of an increasing number of studies since 2009, with the majority being RCTs. Most interventions are delivered face-to-face and rely on trained specialists, limiting their scalability and accessibility. Most have had statistically significant results. However, the included studies demonstrated heterogeneity in terms of delivery, duration, and dose, requiring cautious interpretation of intervention effects. Future research should develop consistent guidelines to standardize the definition of FCR, the measurement tools used, and the timing of follow-up assessments, thereby improving comparability across studies and strengthening the evidence base. Long-term follow-up data are needed to evaluate the sustained effects. As most studies have focused on breast cancer survivors, further research is needed to provide evidence for other cancer populations.

## Supplementary material

10.2196/81579 Multimedia Appendix 1 Search strategies, data dictionary, intervention characteristics, and screening flowchart.

10.2196/81579 Checklist 1 PRISMA-ScR checklist.

## Abbreviations

ACT: acceptance and commitment therapy
CBT: cognitive behavioral therapy
CENTRAL: Cochrane Central Register of Controlled Trials
FCR: fear of cancer recurrence
FoP: fear of progression
MBI: mindfulness-based intervention
PRISMA-ScR: Preferred Reporting Items for Systematic Reviews and Meta-Analyses extension for Scoping Reviews
RCT: randomized controlled trial
