# Supplementary material for: Interventions to Reduce Fear of Cancer Recurrence Among People With Cancer: Scoping Review
Source: JMIR Cancer. 2026 Apr 22;12:e81579. doi: 10.2196/81579 (PMC13102330; doi:10.2196/81579)

# Multimedia Appendix 1. Search strategies, data dictionary, intervention characteristics, and screening flow chart.

Table S1: *Results from searches of the 7 databases* ........ 2
Table S2: *Data dictionary* ........ 6
Table S3: *Timing of Intervention Delivery* ........ 10
Table S4: *Fear of cancer recurrence measurement tool* ........ 11
Table S5*: Characteristics of statistically significant interventions* ........ 14
Figure S1: *Screening flow chart* ........ 31

**Table S1:** *Results from searches of the 7 databases*

- **Medline Search Strategy**

**Search Strategy**: Database(s): Ovid MEDLINE(R) ALL 1980 to April 10, 2024

| Search ID | Search Terms | Results |
|---|---|---|
| 1 | Neoplasms/ | 517743 |
| 2 | (Neoplasms or Neoplas* or cancer or carcinom* or malignant* or tumor* or oncolog*).mp. | 4971105 |
| 3 | 1 or 2 | 4971105 |
| 4 | (Colorectal or colon* or rectal or Bowel or rectum or sigmoid or anal or anus or digestive or Gastrointestinal or Intestinal).mp. | 1743358 |
| 5 | 3 or 4 | 6113086 |
| 6 | ((Fear or anxiety or worr* or concern or distress) adj4 (recurrence or relapse or progress* or reappearance or return)).mp | 5224 |
| 7 | 5 and 6 | 2314 |
| 8 | limit 7 to yr="1980 -Current" | 2309 |

NOTE: [mp=title, abstract, original title, name of substance word, subject heading word, floating sub-heading word, keyword heading word, organism supplementary concept word, protocol supplementary concept word, rare disease supplementary concept word, unique identifier, synonyms]

- **Embase search strategy**

**Search Strategy: Database(s): Ovid Embase ALL 1980 to April 10, 2024**

| **Search ID** | **Search Terms** | **Results** |
|---|---|---|
| **1** | Neoplasms/ | 56676 |
| **2** | (Neoplasms or Neoplas* or cancer or carcinom* or malignant* or tumor* or oncolog*).mp. | 6537715 |
| **3** | 1 or 2 | 6537715 |
| **4** | (Colorectal or colon* or rectal or Bowel or rectum or sigmoid or anal or anus or digestive or Gastrointestinal or Intestinal).mp. | 2421870 |
| **5** | 3 or 4 | 7936446 |
| **6** | ((Fear or anxiety or worr* or concern or distress) adj4 (recurrence or relapse or progress* or reappearance or return)).mp | 8093 |
| **7** | 5 and 6 | 4098 |
| **8** | limit 7 to yr="1980 -Current" | 4094 |

NOTE: [mp=title, abstract, original title, name of substance word, subject heading word, floating sub-heading word, keyword heading word, organism supplementary concept word, protocol supplementary concept word, rare disease supplementary concept word, unique identifier, synonyms]

- **CENTRAL search strategy**

**Between Jan 1980 and Apr 2024**

| **Search ID** | **Search Terms** | **Results** |
|---|---|---|
| #1 | MeSH descriptor: [Neoplasms] explode all trees | 124365 |
| #2 | (Neoplasms or Neoplas* or cancer or carcinom* or malignant* or tumor* or oncolog*):ti,ab,kw (Word variations have been searched) | 275193 |

| | | |
|---|---|---|
| #3 | #1 OR #2 | 287020 |
| #4 | (Colorectal or colon* or rectal or Bowel or rectum or sigmoid or anal or anus or digestive or Gastrointestinal or Intestinal):ti,ab,kw (Word variations have been searched) | 142972 |
| #5 | #3 OR #4 | 380749 |
| #6 | ((Fear or anxiety or worr* or concern or distress) NEAR/4 (recurrence or relapse or progress* or reappearance or return)):ti,ab,kw (Word variations have been searched) | 1687 |
| #7 | #5 AND #6 with Cochrane Library publication date Between Jan 1980 and Apr 2024 | 756 |

- **CINAHL search strategy**

**Search Strategy: Database: CINAHL ALL 1980 to April 10, 2024**

| Search ID | Search Terms | Results |
|---|---|---|
| S1 | MH (MH "Neoplasms") OR ( Neoplasms or Neoplas* or cancer or carcinom* or malignant* or tumor* or oncolog* ) | 904,438 |
| S2 | Colorectal or colon* or rectal or Bowl or rectum or sigmoid or anal or anus or digestive or Gastrointestinal or Intestinal | 249,094 |
| S3 | (Colorectal or colon* or rectal or Bowel or rectum or sigmoid or anal or anus or digestive or Gastrointestinal or Intestinal) AND (S1 OR S2) | 249,094 |
| S4 | (Fear or anxiety or worr* or concern or distress) N4 (recurrence or relapse or progress* or reappearance or return) | 2,902 |
| S5 | ((Fear or anxiety or worr* or concern or distress) N4 (recurrence or relapse or progress* or reappearance or return)) AND (S3 AND S4) Publication Date: 19800101-20241231 | 139 |

- **PsycINFO search strategy**

**Search Strategy: Database: PsycINFO ALL 1980 to April 10, 2024**

| Search ID | Search Terms | Results |
|---|---|---|
| **1** | Neoplasms/ | 45369 |
| **2** | (Neoplasms or Neoplas* or cancer or carcinom* or malignant* or tumor* or oncolog*).mp. [mp=title, abstract, heading word, table of contents, key concepts, original title, tests & measures, mesh word] | 106444 |
| **3** | 1 or 2 | 106444 |
| **4** | (Colorectal or colon* or rectal or Bowel or rectum or sigmoid or anal or anus or digestive or Gastrointestinal or Intestinal).mp. [mp=title, abstract, heading word, table of contents, key concepts, original title, tests & measures, mesh word] | 49836 |
| **5** | 3 or 4 | 148718 |
| **6** | ((Fear or anxiety or worr* or concern or distress) adj4 (recurrence or relapse or progress* or reappearance or return)).mp. [mp=title, abstract, heading word, table of contents, key concepts, original title, tests & measures, mesh word] | 2489 |

| | | |
|---|---|---|
| **7** | 5 and 6 | 832 |
| **8** | limit 7 to yr="1980 -Current" | 830 |

Note: [mp=title, abstract, heading word, drug trade name, original title, device manufacturer, drug manufacturer, device trade name, keyword heading word, floating subheading word, candidate term word

- **The World Health Studies Organization International Clinical Trials Registry Platform （ICTRP）search strategy**

**Search Strategy:**

| **Search ID** | **Search Terms** | **Results** |
|---|---|---|
| 1 | Neoplasms OR Neoplas* OR cancer OR carcinom* OR malignant* OR tumor* OR oncolog* | 38856 records for 34555 trials found |
| 2 | Colorectal OR colon* OR rectal OR Bowel OR rectum OR sigmoid OR anal OR anus OR digestive OR Gastrointestinal OR Intestinal | 8277 records for 7927 trials found |
| 3 | 1 OR 2 | 42975 records for 38578 trials found |
| 4 | (Fear OR anxiety OR worr* OR concern OR distress) AND (recurrence OR relapse OR progress* OR reappearance OR return) | 59 records for 59 trials found |
| 5 | 3 AND 4 | 11 records for 11 trials found |
| 6 | 3 AND 4 with result | 1 records |

- **ProQuest dissertations& thesis global search strategy**

**Search Strategy: Database: ProQuest dissertations& thesis global**

**1980-01-01- 2024—4-10**

| **Search ID** | **Search Terms** | **Results** |
|---|---|---|
| S1 | mainsubject(Neoplasms) OR summary(Neoplasms or Neoplas* or cancer or carcinom* or malignant* or tumor* or oncolog*) OR title(Neoplasms or Neoplas* or cancer or carcinom* or malignant* or tumor* or oncolog*) OR diskw(Neoplasms or Neoplas* or cancer or carcinom* or malignant* or tumor* or oncolog*) | **139,591** |
| S2 | summary(Colorectal or colon* or rectal or Bowel or rectum or sigmoid or anal or anus or digestive or Gastrointestinal or Intestinal) OR title(Colorectal or colon* or rectal or Bowel or rectum or sigmoid or anal or anus or digestive or Gastrointestinal or Intestinal) OR diskw(Colorectal or colon* or rectal or Bowl or rectum or sigmoid or anal or anus or digestive or Gastrointestinal or Intestinal) | **106,769** |
| S3 | [S1] OR [S2] | 230,590 |

| | | |
|---|---|---|
| S4 | summary((Fear or anxiety or worr* or concern or distress) NEAR/4 (recurrence or relapse or progress* or reappearance or return)) OR title((Fear or anxiety or worr* or concern or distress) NEAR/4 (recurrence or relapse or progress* or reappearance or return)) OR diskw((Fear or anxiety or worr* or concern or distress) NEAR/4 (recurrence or relapse or progress* or reappearance or return)) | 1339 |
| S5 | [S3] OR [S4] Limits applied | 237 |

**Table S2:** *Data dictionary*

| Information | Interpretations |
|---|---|
| **General information** | |
| Corresponding author's contact information | Corresponding author's phone number, email address and postal address |
| Author | First name of the author<br>e.g.<br>One author: Smith;<br>Two authors: Smith & Hunt;<br>More than two authors: Smith et al. |
| Year | Online publishing date<br>(format: yyyy) |
| Title | English language and the original Title of the article |
| Journal | Full name of the journal |
| Country/Countries | Country/countries in which the study was conducted.<br>e.g. U.S., UK, Canada, Australia … |
| Study aim | A concise statement outlining the primary goal or objective of a research project |
| **Participant** | |
| Sample size | Total number of participants |
| Age | Mean ± SD or Range |
| Gender | Percentage of participants by gender<br>(e.g. male: female: other options) |
| Diagnosis | Type of participant's cancer type(s) and number<br>e.g., Breast cancer: number |
| Include criteria | Study eligibility criteria, diagnostic criteria of included participants. |

| | |
|---|---|
| Cancer stage | Stages of cancer are classified as Stage I/II/III/IV according to the TNM system. |
| **Concept** | |
| The definition of Fear of recurrence/progression (FCR/FoP) | |
| Who first developed this definition | The full name of the author/ community |
| Study design | Type of study design:<br><br>Randomized controlled trail (RCT): These trials take a homogenous group of study participants and randomly divide them into two separate groups. If the randomization is successful then these two groups should be the same in all respects, both measured confounders and unmeasured factors. The intervention is then implemented in one group and not the other and comparisons of intervention efficacy between the two groups are analysed.<br><br>Pre-post study measures the occurrence of an outcome before and again after a particular intervention is implemented. Pre post studies may be single arm, one group measured before the intervention and again after the intervention, or multiple arms, where there is a comparison between groups. Often there is an arm where there is no intervention. The no-intervention arm acts as the control group in a multiarm pre-post study.<br><br>Non-randomized trial study compare a group where an intervention was performed with a group where there was no intervention.<br><br>Mixed method study: according to the National Institutes of Health, mixed methods strategically integrates or combine rigorous quantitative and qualitative research methods to draw on the strengths of each.<br><br>Non-concurrent multiple baseline design is an experimental design used primarily in applied behavior analysis and other behavioral research fields. It is a type of single-subject experimental design where interventions are introduced at |

| | |
|---|---|
| | different times across multiple baseline conditions. This design is used to assess the effectiveness of interventions while controlling for various confounding factors. |
| When the intervention was applied | e.g. How many months/years after active cancer treatment finished (e.g. during active cancer treatment) |
| Intervention components | Components included in the intervention (e.g., what materials are provided?) |
| Intervention provider | Who delivers the intervention |
| Provider qualifications | Qualifications of the intervention provider (e.g., professional background, training participated in related to the intervention, other) |
| Method of recruitment of participants | How to recruit participants. (e.g., Phone, mail, fliers, other) |
| Intervention frequency | Describe the intervention timing (e.g. (e.g. how many sessions per week?) |
| Intervention intervals | How many days/weeks between sessions |
| Intervention duration | How many weeks for the intervention |
| Definition of control groups (if have) | e.g., no intervention, placebo, minimally active comparator, or components of usual care |
| Duration of follow-up | How long is the follow up period? (e.g., 3 months, 6 months) |
| Theoretical framework of intervention | What is the theoretical framework on which interventions are based? |
| Outcome(s) | Include each outcome |
| Measurement tool | Measurement tools for each outcome |
| Result | Results that are related to scoping review questions (e.g. estimate, Confidence interval and *P* value for each outcome) |
| **Context** | |
| Study setting | Location where interventions was conducted (e.g., hospitals, outpatient, home, community-based |

| | |
|---|---|
| | settings, other) |
| Formats of delivery | Format of intervention delivery (e.g., per support, group-based, individual, face-to-face, digital mental health intervention web-based, app,etc.) |
| **Type of study(methodology)** | e.g., quantitative, mixed methods |
| **key findings** | Key findings that are related to the scoping review questions. |
| **Research gaps** | Limitation of the study |

*Note: If no data write NA

**Table S3:** *Timing of Intervention Delivery*

| When the intervention was applied | Frequency | Percentage (%) |
|---|---|---|
| After diagnosis with cancer but with unclear specific time point | 38 | 31.1 |
| Completed (currently disease-free) or still receiving treatment (no time limit) | 36 | 29.5 |
| Completed primary cancer treatment (at least 6 months) and currently disease-free | 7 | 5.7 |
| Completed primary cancer treatment (at least 1 year) and currently disease-free | 3 | 2.5 |
| Completed primary cancer treatment (at least 5 years) and currently disease-free | 2 | 1.6 |
| Completed primary cancer treatment (within 3 months) and currently disease-free | 5 | 4.1 |
| Completed primary cancer treatment (within 6 months) and currently disease-free | 1 | 0.9 |
| Completed primary cancer treatment (within 1 year) and currently disease-free | 2 | 1.6 |
| Completed primary cancer treatment (within 2 years) and currently disease-free | 5 | 4.1 |
| Completed primary cancer treatment (within 5 years) and currently disease-free | 1 | 0.9 |
| Completed primary cancer treatment (within 15 years) and currently disease-free | 2 | 1.6 |
| Completed primary cancer treatment (between 2 weeks to 2 years) and currently disease-free | 5 | 4.1 |
| Completed primary cancer treatment (between 2 months to 5 years) and currently disease-free | 4 | 3.3 |
| Completed primary cancer treatment (between 3 months to 2.5 years) and currently disease-free | 2 | 1.6 |
| Completed primary cancer treatment (between 3 months to 10 years) and currently disease-free | 9 | 7.4 |

**Table S4:** *Fear of cancer recurrence measurement tool (n=127)*

| Specific Tools | Frequency |
|---|---|
| Fear of cancer recurrence inventory (FCRI) (AhmadiQaragezlou et al 2020 [29]; Ananeva 2020 [63]; Beith et al 2017 [64]; Bin et al 2023 [146]; Burm et al 2019 [39]; Butow et al 2017 [66]; Cohen et azl 2022 [31]; Compen et al 2018 [109]; Dieng et al 2016 [143]; Dieng et al 2019 [145]; Dieng et al 2020 [144]; Dodds et al 2015 [100]; Fisher et al 2019 [67]; Fisher et al 2017 [68]; Gonzalez-Hernandez et al 2018 [101]; Hall et al 2020 [70] ; Kacel 2019 [112]; Lee et al 2022 [113]; Liu et al 2021 [71]; Maheu et al 2023 [72]; Merckaert et al 2017 [73]; Murphy et al 2020 [102]; Nguyen et al 2022 [96]; Russell et al 2019 [118]; Sajadian et al 2021 [121]; Sarizadeh et al 2018 [136]; Savard et al 2018 [46]; Sharpe et al 2019 [47]; Shih et al 2014 [80]; Shumay et al 2013 [132]; Smith et al 2015 [34]; Sun et al 2023 [84]; Tauber et al 2023 [49]; Tomei et al 2014 [79]; Tomei et al 2018 [51]; Tomei et al 2016 [35]; van de Wal et al 2018 [52]; van Helmondt et al 2023 [53]; Wagner et al 2021 [88]; Zhao et al 2023 [55]) | 40 |
| Fear of Cancer Recurrence Inventory-Short Form (FCRI-SF) (Akechi et al 2023 [83]; Brooker et al 2020 [106]; Cillessen et al 2018 [123]; Davidson et al 2018 [30]; Deuning-Smit et al 2024 [40]; Dirkse et al 2020 [90]; Hall et al 2022 [69]; Johns et al 2020 [128]; Luigjes-Huizer et al 2023 [41]; Peng et al 2022 [33]; Smith et al 2020 [76]; Sinclair et al 2023 [133] ; Smith et al 2024 [48]; Subnis 2014 [150]; Tauber et al 2022 [50]; Thewes et al 2012 [78]; Tran et al 2025 [180]; Yoon et al 2023 [151]) | 18 |
| Fears of Cancer Recurrence -7 Item Version (FCR7) **(**Bergerot et al 2023 [104]**;** Bergerot et al 2022 [105]**;** Loughan et al 2021 [141]**;** Loughan et al 2022 [140]**)** | 4 |
| Fears of Cancer Recurrence-4 Item Version (FCR4) (McHale et al 2024 [42]) | 1 |

| | |
|---|---|
| Fear of Progression Questionnaire (FoP-Q) (Hasannezhad Reskati et al 2020 [56]; Herschbach 2012 [93]; Herschbach et al 2010 [92]; Rudolph et al 2018 [137]) | 4 |
| Fear of Progression Questionnaire short form (FoP-Q-SF) (Arch et al 2024 [149]; Frangou et al 2021 [65]; Howells et al 2019 [103]; Li et al 2021 [147]; Neubert et al 2023 [130]; Pradhan et al 2021 [170]; Reb et al 2020 [44]; Reb et al 2020 [45]; Schlecht et al 2023 [74]; Vâlcu et al 2023 [89]; Wang et al 2023 [86]; Weis et al 2020 [181]; Zhao and Xu 2021 [87]) | 13 |
| Concerns about Recurrence Scale (CARS) (Akechi et al 2021 [62]; Akechi et al 2023 [83]; Arch and Mitchell 2016 [134]; Arch et al 2021 [135]; Arch et al 2024 [149]; Fishbein and Arch 2022 [126]; Fishbein et al 2023 [127]; Fishbein et al 2022 [139]; Imai et al 2019 [152]; Lengacher et al 2021 [114]; Lengacher et al 2009 [115]; Lengacher et al 2016 [122]; Lengacher et al 2018 [116]; Lichtenthal et al 2017 [99]; Manne et al 2017 [81]; Momino et al 2017 [82]; Otto 2015 [155]; Park et al 2020 [117]; Sakai et al 2024 [131]; Zhang et al 2022 [142]) | 20 |
| Concerns About Recurrence Questionnaire (CARQ) (Saltbæk et al 2024 [148]) | 1 |
| Cancer Worry Scale (CWS) (Akkol-Solakoglu and Hevey 2023 [61]; Burm et al 2019 [39]; Döking et al 2021 [91]; Eckert et al 2020 [111]; Luberto et al 2019 [124]; Salazar-Alejo et al 2023 [119]; van de Wal et al 2018 [52]; van de Wal et al 2015 [97]; van de Wal et al 2017 [98]; Wang et al 2023 [54]) | 10 |
| Cancer Worry Scale 6 items (CWS-6) (Deuning-Smit et al 2024 [40]) | 1 |
| Worry of Cancer Scale (WOC) (Humphris and Rogers 2012 [94]) | 1 |
| Assessment of Survivor Concerns (ASC) (Chang 2023 [125]) | 1 |
| Fear of cancer recurrence scale (not specified) (Cheng et al 2021 [108]; Crane-Okada et al 2012 [110]; Lebel et al 2014 [32]) | 3 |

| **General tools** | Frequency |
|---|---|
| Personal Questionnaire-Fear of cancer recurrence (PQ-FCR) (Almeida et al 2022 [38]) | 1 |
| Memorial Anxiety Scale for Prostate Cancer (MAX-PC) **(**Chambers et al 2012 [107]**;** Victorson et al 2012 [120]**)** | 2 |
| Quality of life in adult cancer survivors scale (QLACS) (Martin et al 2020 [153]) | 1 |
| Mental Adjustment to Cancer Scale (MINIMAC) (Montesinos et al 2016 [129]) | 1 |
| Functional Assessment of Cancer Therapy-Breast Cancer (FACT-B) **(**Sinclair et al 2020 [75]**;** Ulfig 2020 [36]**;** Ulfig et al 2019 [37]**)** | 3 |
| **Not available (**Sauer and Maatouk 2021 [138]**;** Steinecke et al 2022 [77]**)** | 2 |

**Table S5:** *Characteristics of statistically significant interventions*

| Author & Year | Intervention | sessions | Duration (weeks) | Duration of per session (minutes) | provider(s) | Mode | Follow-up |
|---|---|---|---|---|---|---|---|
| **Ahmadiqaragezlou et al 2020** [29] | Mindfulness-Based Stress Reduction (MBSR) | 8 | 8 | 120 | psychologist | In-person | After intervention |
| **Akechi et al 2023** [83] | Smartphone-Based PST (Kaiketsu-App) and BA (Genki-App) | 8 | 8 | 10 | Researchers | app | 0, 2,4,8 weeks during the study period (weeks 0-8), and the follow-up assessment was conducted at 24 weeks via smartphone. |
| **Almeida et al 2022** [38] | Emotion-focused therapy (EFT) | 13 | 13 | 50-60 | psychologist | in-person | pre-intervention and post -intervention |
| **Ananeva et al 2020** [63] | Conquer Fear (CF) | 5 | 3 | | Psychologist | in-person | baseline and 3 months later |
| **Arch and Mitchell 2016** [134] | Acceptance and Commitment Therapy (ACT) | 7 | 7 | 120 | Psychologist and social worker | in-person | baseline points (3.5, 2, and .5 week[s] prior to the group2 ), mid-intervention |

| | | | | | | | |
|---|---|---|---|---|---|---|---|
| | | | | | | | (Mid), 1 week following the last group session (Post), and 3-months following Post (FU). |
| **Arch et al 2021** [135] | Acceptance and Commitment Therapy (ACT) | 7 | 7 | 120 | Doctors | in-person | baseline, 1, 2, 5, and 8 months post-randomization |
| **Arch et al 2024** [149] | Written exposure-based coping intervention (EASE) | 5 | 5 | 30 | Ph.D. Student | online (zoom) | baseline (Pre), mid-intervention (Mid; after third intervention session), one-week postintervention (Post; 1.5D months postbaseline), 3 months follow-up (FU1; 3 months postbaseline), and 4.5 months follow-up (FU2; 4.5 months postbaseline) |
| **Beith et al 2017** [64] | Conquer Fear (CF) | 5 | 5 | | | in-person | Follow-up assessments occurred immediately, 3- and 6-months |

| | | | | | | | |
|---|---|---|---|---|---|---|---|
| | | | | | | | post-treatment |
| **Bergerot et al 2022** [104] | Mindfulness-Based Cancer Recovery (MBCR) | ≥16 | ≥4 | 20-30 | | online | baseline and 2-weeks and 3 months after intervention |
| **Bergerot et al 2023** [105] | Mindfulness based intervention (MBI) | ≥16 | ≥4 | 20-30 | | online | baseline and 2-weeks and 3 months after intervention |
| **Brooker et al 2020** [106] | Mindful Self-Compassion (MSC) program | 8 | 8 | 105 | psychologist | in-person | After intervention |
| **Burm et al 2019** [39] | Cognitive Behavioral Therapy (CBT) | 12 | 12 | | psychologist | in-person and online | baseline (T0, before randomisation), 3 (T1), 9 (T2) and 15 months (T3) after the baseline assessment |
| **Butow et al 2017** [66] | Conquer Fear (CF) | 5 | 10 | 60-90 | psychologist | in-person | baseline (T0); therapy conclusion (T1); and 3 (T2) and 6 months (T3) later |
| **Chambers et al 2012** [107] | Mindfulness-Based Cognitive Therapy (MBCT) | 8 | 8 | 120 | psychologist | in-person | baseline and 3 months after intervention |
| **Chang et al 2025** [125] | Mindfulness-Based Fitness Training (MBFT) | 8 | 8 | 150 | psychologist | in-person | baseline and after intervention |
| **Cheng et al 2021** | Mindfulness-Based | 5 | 5 | | | online | baseline and 8, 12 |

| | | | | | | | | |
|---|---|---|---|---|---|---|---|---|
| [108] | Fitness Training (MBFT) | | | | | | | and 24 weeks from baseline |
| **Cohen et al 2022** [31] | Mindfulness-Based Cognitive Therapy (MBCT) | 8 | 8 | | 120 | Doctor | in-person | The first MBCT group class, at the conclusion of the 8-week MBCT course,<br>and 6 months after completion of the MBCT intervention |
| **Compen et al 2018** [109] | Mindfulness-Based Cognitive Therapy (MBCT) | 8 | 8 | | 120 | psychologist | in-person and online | baseline and after intervention |
| **Crane-Okada et al 2012** [110] | Mindful Movement Program (MMP) | 12 | 12 | | | psychologist | in-person | 3 time points (baseline, 12 weeks, and 18 weeks) |
| **Davidson et al 2018** [30] | Mini-Adjustment to Fear, Threat<br>and Expectation of Recurrence (Mini-AFTERc) intervention | 1 | 1 | DAY | 30 | 3 clinical nurse specialists | telephone | baseline and postintervention |
| **Deuning-Smit et al 2024** [40] | Cognitive Behavioral Therapy (CBT) | 8 | 8 | | | psychologist | in-person | first session (T0) and after completion of session 8 (T1). |
| **Dieng et al 2016** [143] | melanoma care program | 4 | 4 | | 60 | psychologist | in-person and telephone | baseline, 1 month, and 6 months |
| **Dieng et al 2019** | melanoma care | 4 | 4 | | 60 | psychologist | in-person and | 12 month |

| | | | | | | | |
|---|---|---|---|---|---|---|---|
| [145] | program | | | | | telephone | |
| **Dieng et al 2020** [144] | melanoma care program | 4 | 4 | 60 | psychologist | telephone | 12 month |
| **Dirkse et al 2020** [90] | Cognitive Behavioural Therapy (CBT) | | 8 | | | | |
| **Dodds et al 2015** [100] | Cognitively-Based Compassion Training (CBCT) | 8 | 8 | 120 | Ph.D. social work researcher | in-person | baseline, after intervention and 1 month after |
| **Döking et al 2021** [91] | Cognitive Behavioural Therapy (CBT) | 14 | 14 | | psychologist | in-person and telephone | baseline, postintervention at 4 months , 7-months follow-up, and 14-months follow-up. |
| **Fishbein et al 2023** [127] | Acceptance and Commitment Therapy (ACT) | 7 | 7 | 120 | doctor | in-person | Pre-intervention (Pre, prior to randomization) and Post-intervention (Post). |
| **Fisher et al 2017** [68] | Metacognitive therapy (MCT) | 6 | 6 | 60 | researchers | in-person | 3-months follow-up and in three out of four patients to 6-months follow-up |
| **Frangou et al 2021** [65] | Cognitive Behavioural | 3 | 6 | 90 | psychologist | in-person | 3，6，9，12，15，18 and 24 month |

| | | | | | | | |
|---|---|---|---|---|---|---|---|
| | Therapy (CBT) | | | | | | |
| **Gonzalez-Hernandez et al 2018** [101] | Cognitively-Based Compassion Training (CBCT) | | 8 | | | in-person | |
| **Hall et al 2020** [70] | Mind-body resiliency intervention | 8 | 8 | 90 | researchers | in-person | baseline, postintervention, +1month, and +3 months |
| **Hall et al 2022** [69] | Mind-body resiliency intervention | 8 | 8 | 90 | researchers | in-person | 1 month follow-up, 3 month follow-up |
| **HasannezhadReskati et al 2019** [56] | Acceptance and Commitment Therapy (ACT) | 8 | 8 | 90 | pyschologist | in-person | pre-test, post-test and follow up |
| **Herschbach et al 2010** [92] | Cognitive Behavioural Therapy (CBT) | 4 | 4 | 90 | pyschologist | in-person | initial session (T1), before discharge (T2), as well as 3 months (T3) and 12 months (T4) after discharge |
| **Herschbach et al 2012** [93] | Cognitive Behavioural Therapy (CBT) | 4 | 4 | 90 | pyschologist | in-person | before (T1) and after (T2) the intervention, as well as three (T3) and 12 months (T4) after discharge |
| **Howells et al 2019** [103] | Cognitive Behavioural Therapy (CBT) | 3 | 3 | 90 | | in-person | baseline (T1), posttreatment (T2), 3 months (T3) |

| | | | | | | | |
|---|---|---|---|---|---|---|---|
| **Humphris and Rogers 2012** [94] | AFTER intervention | 6 | 6 | | specialist nurses | in-person | 3, 7, 11 and 15 months following initial treatment |
| **Imai et al 2019** [152] | problem-solving therapy （PST） | 8 | 8 | | doctor and researchers | online (app) | baseline and after-intervention |
| **Johns et al 2020** [128] | Acceptance and Commitment Therapy (ACT) | 6 | 6 | 120 | A doctoral-level researcher | in-person | baseline (T1), after the intervention (T2), 1 month after the intervention (T3), and 6 months after the intervention (T4) |
| **Li et al 2021**[147] | psychological nursing intervention | 5 | 5 | | nurse | in-person | baseline and after-intervention |
| **Lebel et al 2014** [32] | cognitive-existential (CE) group intervention | 6 | 6 | 90 | psychologists, social workers, and nurse | in-person | pre- and immediately post-intervention. and a 3-month follow-up. |
| **Lee et al 2022** [113] | Mindfulness Stress Management t (MSM) intervention | 12 | 12 | 120 | psychologist | in-person | baseline, 0 and 3 months after intervention |
| **Lengacher et al 2009** [115] | Mindfulness-Based Stress Reduction (MBSR) | 6 | 6 | 120 | psychologist | | Baseline, 6 weeks |
| **Lengacher et al 2016** [122] | Mindfulness-Based Stress Reduction (MBSR) | 6 | | 120 | psychologist | in-person | baseline and at 6 and 12 weeks |
| **Lengacher et al 2018** | Mindfulness-Based | 6 | 6 | 120 | psychologist | online | baseline and after |

| | | | | | | | |
|---|---|---|---|---|---|---|---|
| [116] | Stress Reduction (MBSR) | | | | | | intervention |
| **Lengacher et al 2021** [114] | Mindfulness-Based Stress Reduction (MBSR) | 6 | 6 | 120 | psychologist | in-person | Baseline, 6 weeks, 12 weeks |
| **Lichtenthal et al 2017** [99] | Attention and Interpretation Modification for Fear of Breast Cancer Recurrence (AIM-FBCR) | 8 | 4 | 30 | psychologist | in-person | baseline, after intervention, three-month follow-up |
| **Liu et al 2021** [71] | Clinician led Intervention to address Fear of cancer Recurrence (CIFeR) | 5 | 5 | 60 | doctor | in-person | 1 Week and 3 momths |
| **Luberto et al 2019** [124] | Mindfulness-Based Cognitive Therapy (MBCT) | 8 | 8 | 50 | psychologist | in-person | begin of each session |
| **Luigjes-Huizer et al 2023** [41] | Online primary care intervention | 10 | 10 | 30 | psychologist | oline (web/video) | baseline (T0) and four(T1), six (T2) and ten (T3) months after baseline. |
| **Maheu et al 2023** [72] | Fear of cancer recurrence therapy (FORT) | 6 | 6 | 90-120 | clinical psychologists, nurses, and social workers | in-person | baseline (T1), posttreatment (T2), 3 months (T3), and 6 months (T4) posttreatment. |
| **Manne et al 2017** [81] | Supportive counseling (SC) | 8 | 8 | 60 | social workers, | in-person and telephone | T1 = baseline, T2 = |

| | | | | | | | |
|---|---|---|---|---|---|---|---|
| | | | | | master-level or doctoral-level psychologists, or psychiatrists | | 5 weeks after baseline, T3 = 9 weeks, T4 = 6 months, T5 = 12 months, and T6 = 18 months |
| **Martin et al 2020** [153] | iHOPE (Help to Overcome Problems Effectively) intervention | 6 | 6 | 150 | researchers | online (web) | baseline and post program |
| **Merckaert et al 2017** [73] | multiple-component structured manualized group intervention (MGI) | 15 | 24 | 120 | psychologists | in-person | baseline and after-intervention |
| **McHale et al 2024** [153] | Mini-Adjustment to Fear, Threat and Expectation of Recurrence (Mini-AFTERc) intervention | 1 | 1 DAY | 30 | four specialist breast cancer nurse | telephone | 1.5 month and 3 months after intervention |
| **Montesinos and Francisco 2016** [129] | Acceptance and Commitment Therapy (ACT) | 1 | 1 day | 60 | psychologist | in-person | After each intervention and again 3 months later. |
| **Murphy et al 2020** [102] | Cognitive Behavioural Therapy (CBT) | 8 | ≥16 | | online self-managed but clinician supervised | online | baseline， 0 and 3 months after intervention |
| **Nguyen et al 2022** | virtual telephone | | | | pyschologist | online and | pre-intervention |

| | | | | | | | |
|---|---|---|---|---|---|---|---|
| [96] | coaching program | | | | | telephone | (baseline) and post-intervention (6-month and 12-month time points) |
| **Otto 2015** [155] | Gratitude intervention | 6 | 6 | | researchers | Onli24e （web） | Immediately after completing the sixth weekly survey, participants (Post); 1 month (FU1) and 3 months (FU2) following the sixth weekly survey. |
| **Park et al 2020** [117] | Mindfulness-Based Cognitive Therapy (MBCT) | 8 | 8 | 120 | clinical psychologists, psychiatrists, and nurses | In-person | baseline, 8,12 weeks after intervention |
| **Peng et al 2022** [33] | Mindfulness based intervention (MBI) | 6 | 6 | 90 | psychologist | online | baseline, postintervention, 1-month postintervention |
| **Reb et al 2020** [44] | Conquer Fear (CF) | 7 | 8 | 60.7 | nurses | online, telephone and in-person | 8, 12 weeks after intervention |
| **Reb et al 2020** [45] | Day-by-Day (DBD) intervention | 7 | 8 | 45 | two advanced practice nurses (APNs),the principal | online (web) | baseline and postintervention; 8 weeks, 12 weeks |

| | | | | | | | |
|---|---|---|---|---|---|---|---|
| | | | | | investigator (PI), a psychologist, and a education specialist | | |
| **Rudolph et al 2018** [137] | Acceptance and Commitment Therapy (ACT) | 6 | 6 | 90 | Psychologist | in-person | pre-intervention and post -intervention |
| **Russell et al 2019** [118] | Mindfulness based intervention (MBI) | 6 | 6 | | | online | baseline and after intervention |
| **Sajadian et al 2021** [121] | Psycho-spiritual therapy | 11 | 11 | 30 | psychologist | in-person | pre-intervention and post -intervention |
| **Sakai et al 2022** [131] | Acceptance and Commitment Therapy (ACT) | 17 | 30 | 50 | | in-person aka online | pre-intervention and post -intervention |
| **Salazar-Alejo et al 2023** [119] | Mindfulness-Based Stress Reduction (MBSR) | 8 | 8 | | psychologist | online | baseline, 2 months, 5 months, and 8 months after intervention |
| **Saltbaek et al 2024** [148] | Nurse-Led Individualized Follow-Up | 3-5 | 24 | 60 | nurse | in-person | 0, 3, 6, 9, 12, 18, 24, 30, and 36 months after random assignment |
| **Sarizadeh et al 2018** [136] | Acceptance and Commitment Therapy (ACT) | | | | psychologist | in-person | before and after intervention |
| **Sauer and Maatouk 2021** [138] | Acceptance and Commitment | 8 | 8 | | psychologist | in-person | pre-intervention and post |

| | | | | | | | |
|---|---|---|---|---|---|---|---|
| | Therapy (ACT) | | | | | | -intervention |
| **Savard et al 2018** [46] | Cognitive Behavioural Therapy (CBT) | 5 | 5 | | psychologists | in-person | baseline and posttreatment |
| **Schlecht et al 2023** [74] | Video-Based Intervention | | 16 | 12-30 | psychologist | online (video) | after the intervention group finished the video intervention, and when the control group ended their video intervention ); 3 months from the end of the video intervention of each group |
| **Sharpe et al 2019** [47] | Conquer Fear (CF) | 5 | 5 | 60-90 | three psychologists | in-person | before and after intervention and at 6 months' follow-up |
| **Shih et al 2014** [80] | Conquer Fear (CF) | 5 | 5 | | psychologist or psychiatrist | in-person | baseline, after intervention, two-month follow-up |
| **Sinclair et al 2020** [75] | Acceptance and Commitment Therapy (ACT) and patient education intervention | 6 | 6 | | psychologist | in-person | before, after and 12 weeks upon completion of the intervention |
| **Sinclair et al 2023** [133] | Acceptance and Commitment | 6 | 6 | 120 | pyschologist | in-person | prior to starting (T1), after |

| | | | | | | | |
|---|---|---|---|---|---|---|---|
| | Therapy (ACT) | | | | | | completion (T2) and 12 weeks following group programme participation (T3). |
| **Smith et al 2020** [76] | cognitive behavioural, acceptance and commitment, metacognitive and relaxation therapy | 6 | | | Researchers | Webiste | T1 (baseline), T2 (end of intervention), and T3 (3-month follow-up). |
| **Smith et al 2015** [34] | Conquer Fear (CF) | 5 | 5 | 60-90 | a trained psychologist | in-person | baseline, immediately post-intervention, and 2 months later. |
| **Smith et al 2024** [48] | Conquer Fear (CF) | 10 | 22 | | researchers | online（web) | baseline (T0);e post-intervention questionnaires 10 weeks (T1) and 22 weeks (T2) post-baseline |
| **Steinecke et al 2022** [77] | Psychological group therapy program | | | | 2 psychologists | in-person | pre-intervention and post -intervention |
| **Subnis 2014** [150] | Online expressive writing intervention(EW) | 4 | 4 days | 20-30 | psychologist | Website | Days 2, 7 and 49 |
| **Sun et al 2023** [84] | Traditional Chinese Medicine (TCM) | 6 | 6 | 90 | TCM oncologist | online | baseline and after-intervention |

| | | | | | | | |
|---|---|---|---|---|---|---|---|
| | interventions combined group psychotherapy-Mindfulness and Cognitive behavioral therapy | | | | and psychiatrist | | |
| **Tauber et al 2022** [50] | Conquer Fear (CF) | 6 | 6 | 30 (1 session); 120 (5 sessions) | psychologist | in-person | baseline, post-treatment, and at 3 and 6 months follow-up |
| **Tauber et al 2023** [49] | Conquer Fear (CF) | 6 | 6 | 30 (1 session); 120 (5 sessions | psychologist | in-person | baseline (T1), 1 week post-intervention (T2), three (T3) and six (T4) months later |
| **Thewes et al 2012** [78] | Conquer Fear (CF) | 5 | 5 | | Eight experienced clinical psychologists and one psychiatrist | in-person | pre-treatment, at treatment completion and 2 months post-treatment. |
| **Tomei et al 2016** [35] | cognitive-existential (CE) psychotherapy intervention | 6 | 6 | 60-90 | researchers | in-person ome online | a 4-week baseline period and throughout the 6-week intervention |
| **Tomei et al 2018** [51] | cognitive-existential (CE) psychotherapy intervention | 6 | 6 | 60-90 | clinical psychologists | in-person and online | before the intervention (T1), after the intervention (T2), |

| | | | | | | | |
|---|---|---|---|---|---|---|---|
| | | | | | | | and at 3-month follow-up (T3). |
| **Tran et al 2025** [180] | Fear-Less self-management intervention (stepped-care framework) | 5 | 5 | | doctor | in-person | Re-screening and evaluation measures were completed after five weeks. |
| **Ulfig et al 2019** [37] | Cognitive Behavioural Therapy (CBT) | 6 | 6 | 90 | graduate student | in-person | (T0: Week 0); s (T1: Week 7); (T2: Week 14); (T3: Week 21); (T4: Week 72); |
| **Ulfig et al 2020** [36] | Cognitive Behavioural Therapy (CBT) | 6 | 6 | 90 | graduate student | in-person | (T0: Week 0); s (T1: Week 7); (T2: Week 14); (T3: Week 21); (T4: Week 72); |
| **VanDeWal et al 2015** [97] | Blended Cognitive Behavioural Therapy (CBT) | 6 | 12 | | psychologist | in-person and online | baseline, postintervention at 6 months, 12-months follow-up |
| **vandeWal et al 2017** [98] | Blended Cognitive Behavioural Therapy (bCBT) | 5 | 12 | 75 | pyschologist | in-person and online | baseline (T0) and 3 months later (T1) |
| **vandeWal et al 2018** [52] | Cognitive Behavioural Therapy (CBT) | 9 | 12 | 90 (first session); 45 other sessions | pyschologist | in-person and online | baseline, postintervention at 6 months , 12-months follow-up, |

| | | | | | | | |
|---|---|---|---|---|---|---|---|
| **Valcu et al 2023** [89] | Cognitive Behavioural Therapy (CBT) | 12 | 12 | 150 | psychologist | videoconference | pre-intervention and post -intervention |
| **Victorson et al 2012** [120] | Mindfulness based stress reduction (MBSR) | 8 | 8 | | psychologist | in-person | month 0 to month 6 and 12 |
| **Wagner et al 2021** [88] | FoRtitude | 12-16 | 4 | 5-20 | | online | baseline (T0) and at 4 (T1) and 8 (T2) weeks after the first FoRtitude site log-in |
| **Wang et al 2023** [54] | Managing cancer and living meaningfully (CALM) | 6 | 24 | 45-60 | psychologist | in-person | baseline (T0) and after two (T1), four (T2), and six (T3) intervention sessions. |
| **Wang et al 2023** [86] | Meaning-centered group psychotherapy (MCGP) | 8 | 8 | 120 | psychologist | in-person | baseline and postintervention |
| **Weis et al 2019** [181] | Psychoeducational (PE) group intervention. | 6 | 6 | 120 | psychologist | in-person | baseline (T1), at the end of the intervention after 6 weeks (T2), and 6 weeks after (T3) |
| **Zhao and Xu 2021** [87] | Cognitive Behavioural Therapy (CBT) | | 12 | | neurosurgeon, one clinical rehabilitation therapist, psychologist and nurses | In-person | baseline and after-intervention |

| | | | | | | | |
|---|---|---|---|---|---|---|---|
| **Zhao et al 2023** [55] | Managing cancer and living meaningfully (CALM) | 6 | 6 | 30 | graduate students, oncologist and psychologist | in-person | immediately (T1), 2 months (T2), and 4 months (T3) after treatment |

Figure S1: *Screening flow chart*

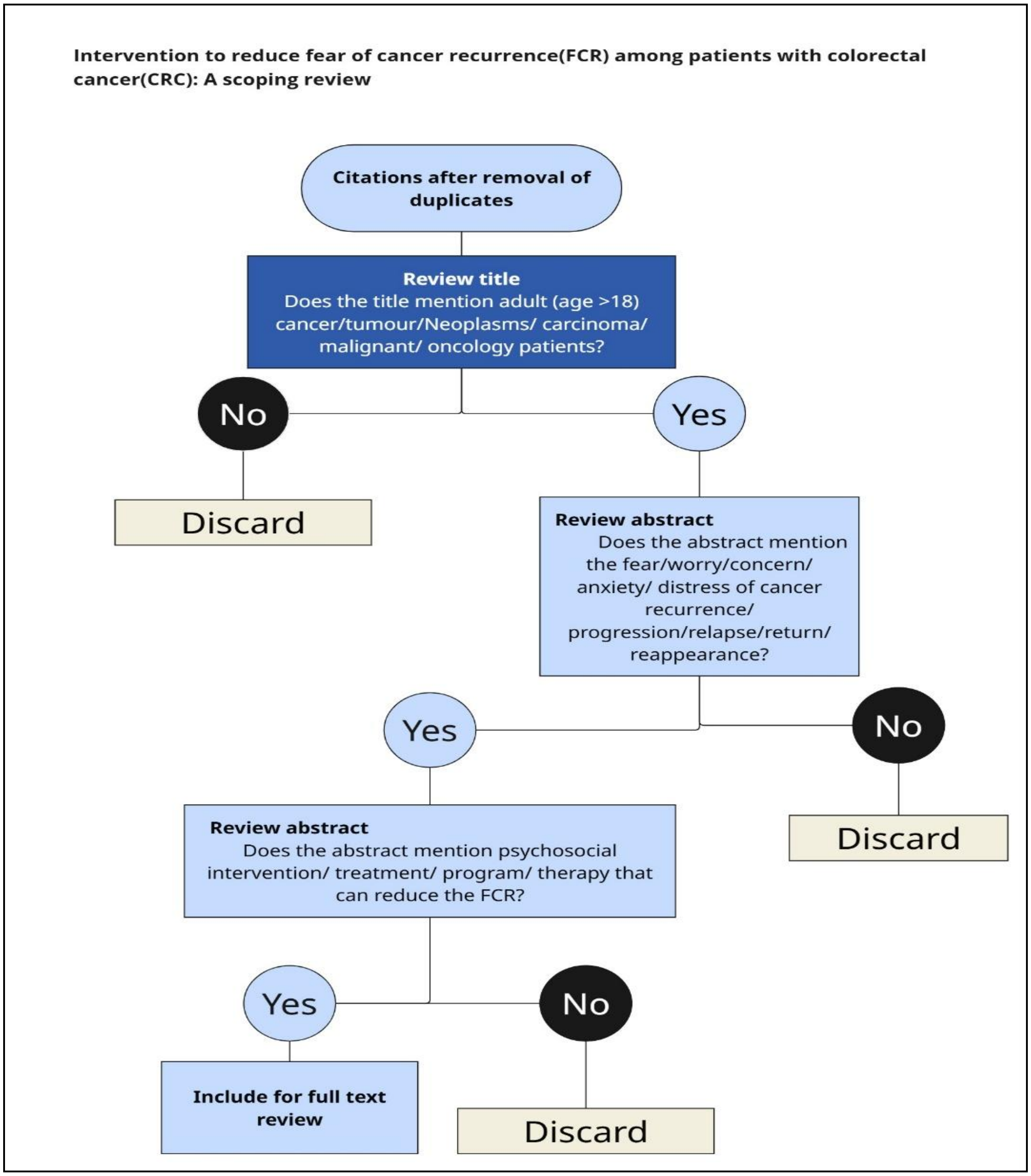

Supplement: Multimedia Appendix 1 [file cancer-v12-e81579-s001.pdf]
